# Effects of depression on employment and social outcomes: a Mendelian randomisation study

**DOI:** 10.1136/jech-2021-218074

**Published:** 2022-03-22

**Authors:** Desmond Campbell, Michael James Green, Neil Davies, Evangelia Demou, Laura D Howe, Sean Harrison, Daniel J Smith, David M Howard, Andrew M McIntosh, Marcus Munafò, Srinivasa Vittal Katikireddi

**Affiliations:** 1 MRC/CSO Social and Public Health Sciences Unit, Institute of Health and Wellbeing, University of Glasgow, Glasgow, UK; 2 MRC Integrative Epidemiology Unit (IEU), Population Health Sciences, Bristol Medical School, University of Bristol, Bristol, UK; 3 K.G. Jebsen Center for Genetic Epidemiology, Department of Public Health and Nursing, NTNU, Norwegian University of Science and Technology, Trondheim, Norway; 4 Division of Psychiatry, Centre for Clinical Brain Sciences, Royal Edinburgh Hospital, University of Edinburgh, Edinburgh, UK; 5 Social, Genetic and Developmental Psychiatry Centre, Institute of Psychiatry, Psychology & Neuroscience, King's College London, London, UK; 6 School of Psychological Science, University of Bristol, Bristol, UK

**Keywords:** Keywords

## Abstract

**Background:**

Depression is associated with socioeconomic disadvantage. However, whether and how depression exerts a causal effect on employment remains unclear. We used Mendelian randomisation (MR) to investigate whether depression affects employment and related outcomes in the UK Biobank dataset.

**Methods:**

We selected 227 242 working-age participants (40–64 in men, 40–59 years for women) of white British ethnicity/ancestry with suitable genetic data in the UK Biobank study. We used 30 independent genetic variants associated with depression as instruments. We conducted observational and two-sample MR analyses. Outcomes were employment status (employed vs not, and employed vs sickness/disability, unemployment, retirement or caring for home/family); weekly hours worked (among employed); Townsend Deprivation Index; highest educational attainment; and household income.

**Results:**

People who had experienced depression had higher odds of non-employment, sickness/disability, unemployment, caring for home/family and early retirement. Depression was associated with reduced weekly hours worked, lower household income and lower educational attainment, and increased deprivation. MR analyses suggested depression liability caused increased non-employment (OR 1.16, 95% CI 1.06 to 1.26) and sickness/disability (OR 1.56, 95% CI 1.34 to 1.82), but was not causal for caring for home/family, early retirement or unemployment. There was little evidence from MR that depression affected weekly hours worked, educational attainment, household income or deprivation.

**Conclusions:**

Depression liability appears to cause increased non-employment, particularly by increasing disability. There was little evidence of depression affecting early retirement, hours worked or household income, but power was low. Effective treatment of depression might have important economic benefits to individuals and society.

## Introduction

Depression is a leading cause of disability, estimated to affect 300 million people worldwide, and to have increased in prevalence by 18% in the decade to 2015.^1^ Due to its prevalence and debilitating nature, understanding the relationship between depression and adverse socioeconomic outcomes has important policy implications for the allocation of government resources.

Associations between socioeconomic disadvantage and depression have long been observed.^2^ The extent to which these represent causal impacts of depression on socioeconomic disadvantage (referred to as health selection) is unclear. Alternative explanations include socioeconomic disadvantage causing depression (social causation) and confounding factors causing both depression and socioeconomic disadvantage (indirect selection).^3–7^ Disentangling the contributions of these explanations to observed associations is difficult.^3 4^ Confounding can be challenging to address, given the difficulty of accurately measuring socioeconomic variables across the life course.

Mendelian randomisation (MR) is an instrumental variable approach which estimates an exposure’s effect on outcomes using exposure-associated genetic variants as instrumental variables.^8^ This differentiates MR from observational analyses which rely on assumptions of no unmeasured confounding of the exposure–outcome relationship. Instead, MR relies on an assumption of no unmeasured confounding of the genetic variant–outcome relationship. Use of genetic instruments mitigates reverse causation concerns. MR estimates the causal effect of a lifelong tendency to an exposure, rather than short-term effects.^9^

We employed two-sample MR to investigate the health selection hypothesis. We estimated the effect of depression liability on employment outcomes (non-employment, sickness/disability, early retirement, caring for home/family and unemployment) and on socioeconomic outcomes (household income, hours worked (among the employed), educational attainment and area-based deprivation) in the UK Biobank dataset. We tested for sex differences in all effects. We compared the MR estimates to multivariable adjusted regression estimated associations of depression and outcomes.

## Methods

### Study population

The UK Biobank study collected data on half a million individuals aged 40–69 from across mainland Britain (2006–2010).^10^ Participants were excluded from the current study if (1) they were not of working age (ie, above retirement age at assessment time: 60 years for women, 65 years for men); (2) they self-reported ethnicity other than white British; (3) their genetically determined ancestry did not match their self-report; (4) they had withdrawn from the study; (5) they were overly genetically related; (6) there were issues with their genetic data; or (7) they were missing all investigated outcomes. Exclusions are detailed in sections 1 and 2 and in the Strengthening the Reporting of Observational Studies in Epidemiology (STROBE) flowchart (online supplemental figure S1).

10.1136/jech-2021-218074.supp1 Supplementary data

**Figure 1:**
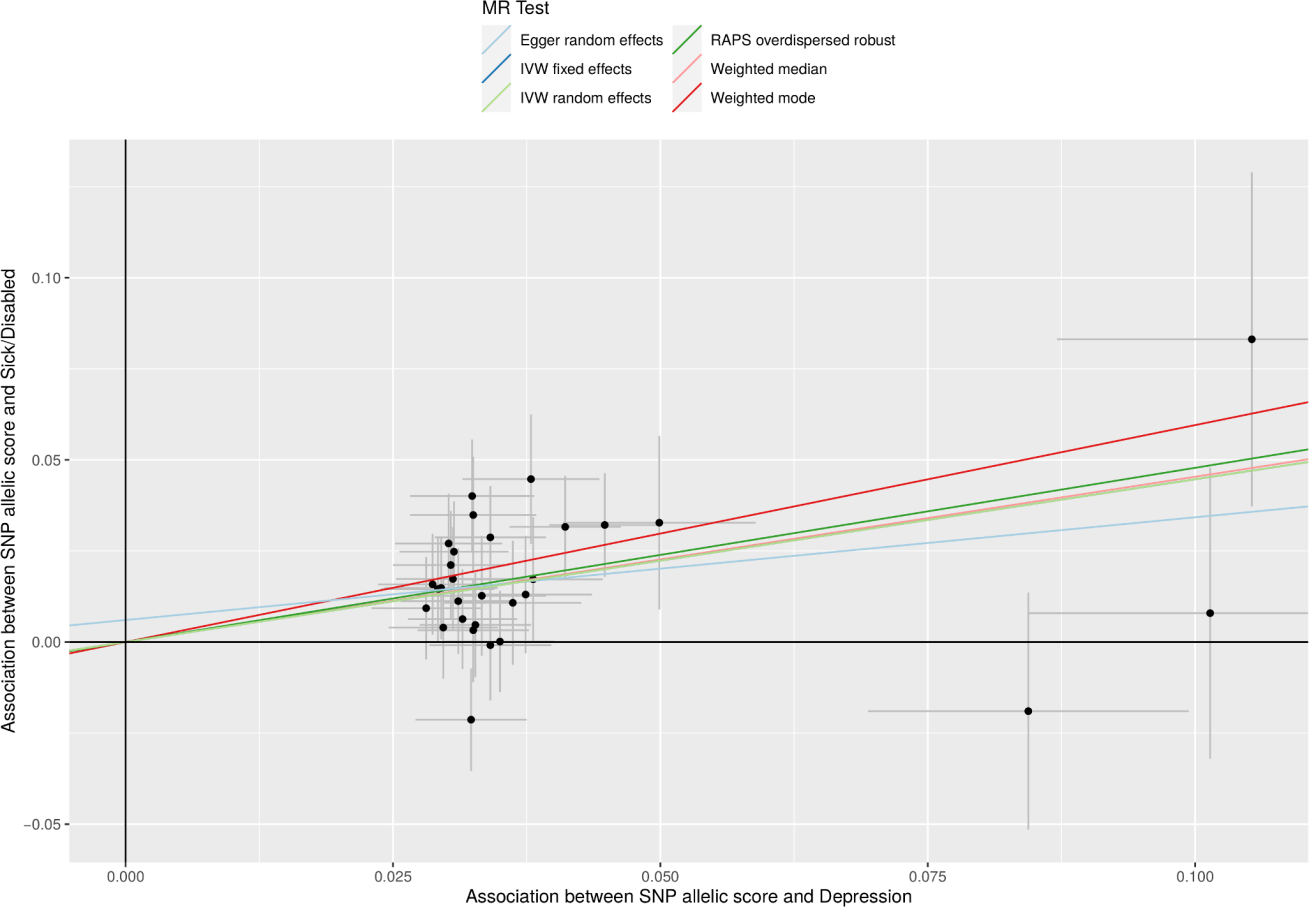
Scatter plot of sick/disabled–SNP associations versus exposure–SNP associations. X axis includes depression–SNP regression coefficient estimates from Howard and colleagues^11^; Y axis includes sick/disabled–SNP log odds from UK Biobank regressions. Also plotted are the fits for several causal effect estimation methods. MR, Mendelian randomisation; RAPS, Robust Adjusted Profile Score; SNP, single-nucleotide polymorphism.

### Depression single-nucleotide polymorphisms (SNPs)

A recent genome-wide association study reported SNPs associated with depression.^11^ From the authors we obtained association results excluding the UK Biobank cohort (ie, based on the Psychiatric Genomics Consortium and 23andMe cohorts alone). From these we identified associated SNPs (p value ≤5×10^−8^). SNPs were excluded based on the Hardy-Weinberg equilibrium (family-wise error rate <1), information content (info score <0.9), minor allele frequency (MAF <0.01) and being palindromic with a high MAF (MAF >0.4). Clumping of remaining SNPs (Physical distance threshold for clumping=10000 kb, R^2^=0.01), yielded a subset of 30 independent associated SNPs, our instrument SNP set. For further details, see section 2 and online supplemental figure S2 and table S11.

**Figure 2:**
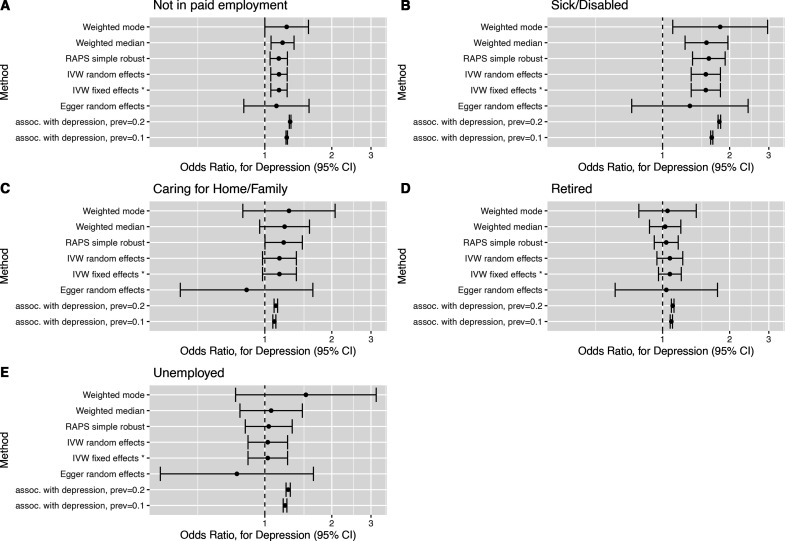
Forest plots of causal effect estimates of depression on employment outcome. Causal effect estimates for change in depression affection status from unaffected to affected on (A) not in paid employment, (B) sick/disabled, (C) caring for home/family, (D) early retirement and (E) unemployment. The association estimates transformed onto the same scale as the Mendelian randomisation estimates are presented in rows prefixed ‘assoc. with depression’, for example, ‘assoc. with depression, prev=0.1’, where prev=0.1 indicates a baseline depression prevalence of 10%. RAPS, Robust Adjusted Profile Score.

### Outcomes

The outcomes used were previously described in our paper relating body mass index to employment outcomes.^12^ All outcomes were obtained at the baseline interview. Current employment status was self-reported, with the five most common categories being (1) ‘in paid employment or self-employed’; (2) retired; (3) sickness/disability (ie, not working due to health); (4) caring for home/family; and (5) unemployed. As the sample only included those of working age, anyone retired was in early retirement. For brevity, we will refer to in paid employment or self-employed as being in paid employment throughout. Employment status was recoded into (1) a binary variable contrasting all other categories (hereafter referred to as non-employment) against being in paid employment and (2) into four binary variables comparing each other category against being in paid employment. Where respondents endorsed multiple categories (<8% participants), ‘employed’ took priority in coding the binary variables. We also considered self-reported weekly hours in paid employment, Townsend Deprivation Index (TDI), household income and highest educational attainment as outcomes. TDI is a measure of area-based deprivation^13^; greater TDI scores imply greater deprivation. Highest educational attainment was an ordinal variable coding for UK academic qualifications from lowest to highest: (1) none of the below; (2) Certificates of Secondary Education (CSEs) or equivalent; (3) O levels/General Certificates of Secondary Education (GCSEs) or equivalent; (4) A levels/AS levels or equivalent; (5) National Vocational Qualification (NVQ) or Higher National Diploma (HND) or Higher National Certificate (HNC) or equivalent; (6) other professional qualifications, for example, nursing and teaching; and (7) college or university degree. Annual gross household income was coded as an ordinal variable: (1) less than £18 000, (2) £18 000–£30 999, (3) £31 000–£51 999, (4) £52 000–£100 000 and (5) greater than £100 000. Table 1 summarises these outcomes. For details of UK Biobank variables used, see section 2 and online supplemental table S10.

**Table 1:** Study sample characteristics

	Female	Male	Overall
Sample size, n (%)	104945 (45.5)	125846 (54.5)	230791 (100)
Age, mean (SD)	50.96 (5.55)	54.46 (7.04)	52.87 (6.64)
Depression, n (%)	17676 (16.8)	15604 (12.4)	33280 (14.4)
Polygenic Risk Score, mean (SD)	26.03 (3.41)	26.08 (3.41)	26.06 (3.41)
Employment Category, n (%)			
In paid employment	83292 (79.4)	90303 (71.8)	173595 (75.2)
Not in paid employment	20791 (19.8)	34507 (27.4)	55298 (24.0)
Early retirement	6768 (6.4)	24036 (19.1)	30804 (13.3)
Sick/disabled	4978 (4.7)	6848 (5.4)	11826 (5.1)
Caring for home/family	7534 (7.2)	1080 (0.9)	8614 (3.7)
Unemployed	1640 (1.6)	3558 (2.8)	5198 (2.3)
NA	20013 (19.1)	31985 (25.4)	51998 (22.5)
Hours worked weekly, mean (SD)	32.28 (11.60)	40.34 (11.07)	36.47 (12.02)
Townsend Deprivation Index, mean (SD)	−1.49 (2.93)	−1.48 (3.02)	−1.48 (2.98)
Household Income, n (%)			
Less than 18,000	13707 (13.1)	18055 (14.3)	31762 (13.8)
18,000 to 30,999	19968 (19.0)	25266 (20.1)	45234 (19.6)
31,000 to 51,999	28012 (26.7)	33279 (26.4)	61291 (26.6)
52,000 to 100,000	24485 (23.3)	29693 (23.6)	54178 (23.5)
Greater than 100,000	6303 (6.0)	8030 (6.4)	14333 (6.2)
NA	12470 (11.9)	11523 (9.2)	23993 (10.4)
Highest educational attainment, n (%)			
None of the below	9321 (8.9)	17023 (13.5)	26344 (11.4)
CSEs or equivalent	5709 (5.4)	5035 (4.0)	10744 (4.7)
O levels/GCSEs or equivalent	15486 (14.8)	12937 (10.3)	28423 (12.3)
A levels/AS levels or equivalent	6896 (6.6)	6562 (5.2)	13458 (5.8)
NVQ or HND or HNC or equivalent	13834 (13.2)	20850 (16.6)	34684 (15.0)
Other professional qualifications e.g. nursing, teaching	15518 (14.8)	18354 (14.6)	33872 (14.7)
College or University degree	37508 (35.7)	44076 (35.0)	81584 (35.3)
NA	673 (0.6)	1009 (0.8)	1682 (0.7)

Employment category: respondents could endorse multiple categories. The ‘not in paid employment’ category is a composite of the listed non-employment categories. The ‘in paid employment’ and ‘not in paid employment’ sum to less than 100% due to some participants giving invalid answers for the employment question. The numbers in this table are the same as in our previous body mass index employment study.^12^

NA, not applicable.

### Exposure

A dichotomous indicator variable for depression was created, indicated by a hospital inpatient International Classification of Diseases, 9th Revision (ICD-9), or International Classification of Diseases, 10th Revision (ICD-10), depression diagnosis, self-reported depression or self-report of seeing a psychiatrist for depression, anxiety or tension (see online supplemental section 2). Prevalence of this phenotype in participants was 12.4% and 16.8% in men and women, respectively. This phenotype was used for association analyses and Polygenic Risk Score (PRS) regression. The MR analyses estimates relate to the Howard *et al* 11 depression phenotype.

### Statistical analyses

We generated an unweighted PRS for depression for each participant, calculated as the number of risk alleles carried across all instrument SNPs. To confirm the depression–instrument SNPs relationship in our sample, we regressed depression on PRS adjusting for age, sex, study assessment centre and 40 genetic principal components (GPCs). Inclusion of GPCs (and study centres) as covariates in a regression is a standard way of correcting for confounding between genes and outcomes (population stratification). We used all 40 GPCs, available from UK Biobank, as covariates. Although some GPCs may be redundant, their inclusion does no harm other than reducing power. For details, see online supplemental section 2.

We investigated the multivariable adjusted association of depression with outcomes. Regression models were fitted adjusting for age, sex, study assessment centre and 40 GPCs. For household income, an additional covariate, number in household (values were winsorised to 12), was added to the regression. As this covariate strongly predicted household income, it was carried forward into subsequent MR analyses of household income.

To facilitate comparison of the MR and association study estimates, the estimate for the regression of each outcome on depression was transformed onto the same scale as the MR estimates. Major depression has a prevalence of about 15%, and women are at twice the risk as men. Therefore, depression prevalences of 10% and 20% are appropriate for men and women, respectively.^14^ These prevalences were used in transforming the association results onto the MR scale. The transformation used is detailed in online supplemental section 2.

We estimated causal relationships via two-sample MR, the inputs for which were SNP–exposure associations (obtained from Howard *et al*), and SNP–outcome associations (from our sample). SNP–outcome associations were estimated using linear, logistic and ordinal regressions of continuous, binary and ordinal outcomes on each SNP, adjusting for age, sex, study assessment centre, and 40 GPCs (implemented using PLINK V.1.9).^15^ The MR causal effect estimates obtained gave predicted outcome change in response to depression prevalence increasing by one unit on the log odds scale.^16^

We estimated the causal effect for depression liability on outcomes using methods available in the R package TwoSampleMR.^17^ We used the Rücker model selection framework to identify the best fitting model from fixed and random effect versions of the inverse-variance weighted (IVW) and Egger methods.^18 19^ However, the various methods each have their own strengths and weaknesses and their estimates should be considered together. IVW and Egger methods do not allow for some SNPs being outliers from their respective models. In contrast, the median and mode-based MR methods allow a high proportion of SNPs to be invalid instruments under balanced pleiotropy. The simple median method has a 50% breakdown level; that is, it provides a consistent estimate of the causal effect as long as at least 50% of genetic variants are valid instruments. The weighted median method provides a consistent estimate if >50% of the weight is on valid instruments. It is biased under directional pleiotropy (though not as badly as IVW) because the median effect (even when that of a valid instrument) will tend towards that of the directional pleiotropy SNPs. A penalised median method reduces such bias further.^20^ Mode methods are similar to the median methods except that the mode of a smoothed approximation of the distribution of SNP causal effect estimates is used in place of the median. The mode method has a higher (though unknown) breakdown level than the median method.^21^ The Robust Adjusted Profile Score method uses profile likelihood to model weak instruments and balanced pleiotropy, and is robust to idiosyncratic pleiotropy in a small proportion of outliers by capping their influence using a Huber/Tukey loss function.^22^

To assess potential MR assumption violations, we estimated causal effects using a wide range of MR estimators. We tested for heterogeneity in causal effect estimates and conducted test for unbalanced horizontal pleiotropy. We calculated $I_{GX}^{2}$ , a measure of the degree of violation of the no measurement error (NOME) assumption for SNP–exposure associations.^23^ To assess whether any single SNP was driving effect estimates, we conducted single SNP MR analyses and leave one SNP out MR analyses. We attempted to repeat analyses with overly influential SNPs removed, but no overly influential SNPs were identified. P values are reported without multiple testing correction.^24^ Details are provided in online supplemental section 2.

To investigate sex differences in the impacts of depression, MR analyses were repeated, stratified by sex. Wald tests were used to test for effect size difference between the sexes. The impact of sex on the genetic architecture of depression has been investigated and while modest differences have been found, there is little support for major differences in genetic architecture across sexes.^25^ We found there was little difference between the sexes in the distribution of the PRS (see table 1). We repeated our depression PRS regression with the addition of a PRS×sex interaction term. The inclusion of the interaction term was not supported (interaction term p value=0.91). In light of these considerations, we decided to use our main MR analysis SNP set for our sex-specific MR analyses, rather than the less powerful sex-specific SNP sets. For further details, see online supplemental section 2.

## Results

The sample (see table 1) comprised 230791 genetically unrelated working-age white British participants. The majority (54.5%) were men. Men tended to be older, worked more hours weekly, reported early retirement more frequently and being in paid work and caring for home and family less frequently.

Results for the regression of outcomes on depression are presented in table 2. People who experienced depression had higher odds of reporting not being in paid employment (OR 2.27, 95% CI 2.21 to 2.33) and being in each constituent non-employment category, especially being sick/disabled (OR 6.27, 95% CI 6.02 to 6.53). They also had lower weekly hours in paid employment (−1.09 hours, 95% CI −1.25 to –0.93) and higher TDI (ie, were more deprived) (beta 0.80, 95% CI 0.76 to 0.83). Ordinal regressions found depression associated with reduced household income level (OR 0.53, 95% CI 0.51 to 0.54) and lower educational attainment (OR 0.83, 95% CI 0.81 to 0.85). Results for covariates are shown in online supplemental table S1.

**Table 2:** Association of outcomes with depression

Employment category	OR	95% CI	P value	Complete obs (N)	OR (MR scale)	95% CI (MR scale)
Not in paid employment	2.27	2.21 to 2.33	0.0E+00	228 893	1.28	1.27 to 1.29
Sick/disabled	6.27	6.02 to 6.53	0.0E+00	185 421	1.74	1.72 to 1.76
Caring for home/family	1.43	1.35 to 1.51	2.2E-34	182 209	1.11	1.09 to 1.13
Retired	1.38	1.33 to 1.44	2.3E-51	204 399	1.10	1.09 to 1.12
Unemployed	2.13	1.99 to 2.28	4.3E-104	178 793	1.26	1.23 to 1.28
**Outcome**	**Beta**	**95% CI**	**P value**	**Complete obs (N)**	**Beta (MR scale)**	**95%** **CI (MR scale)**
Townsend Deprivation Index	0.80	0.765 to 0.83	0.0E+00	230 474	0.24	0.231 to 0.25
Hours worked	−1.09	−1.25 to to 0.93	2.8E-39	171 618	−0.33	−0.38 to –0.28
Number in household	−0.23	−0.25 to 0.22	2.3E-208	229 857	−0.07	−0.074 to –0.065
**Outcome**	**OR**	**95%** **CI**	**P value**	**Complete obs (N)**	**OR (MR scale)**	**95%** **CI (MR scale)**
Highest educational attainment	0.83	0.815 to 0.85	2.2E-65	229 041	0.95	0.94 to 0.952
Household income	0.53	0.515 to 0.54	0.0E+00	206 547	0.82	0.819 to 0.83

All associations are adjusted for age, sex, study assessment centre, and genetic principal components. Results for household income were obtained with or without additional adjustment for number in household (winsorised to 12). These results were not qualitatively different, results with adjustment are reported. Associations transformed onto the MR scale (columns suffixed ‘(MR Scale)’) assumed a depression prevalence of 15%

The depression OR for each unit increase in PRS was 1.02 (95% CI 1.017 to 1.024). The PRS had a delta Akaike information criterion (AIC) of 133.6 (relative likelihood=9.7e-30), confirming that the instrument SNP set was strongly associated with depression (online supplemental table S2).

Figure 1 plots the SNP–outcome against SNP–exposure associations for the sick/disabled outcome. Consistent with the MR analysis assumptions, SNPs more strongly associated with exposure (depression) were also more strongly associated with the outcome. Similar plots are available for the other outcomes (eg, online supplemental figure S9).

**Figure 3:**
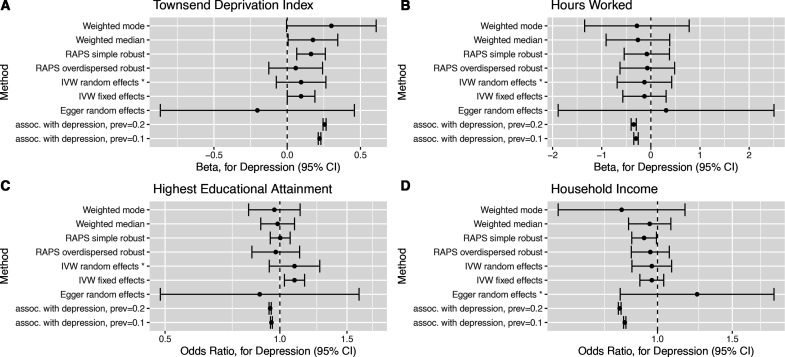
Forest plots of causal effect estimates of depression on other outcomes. Causal effect estimates for change in depression affection status from unaffected to affected on (A) Townsend Deprivation Index, (B) hours worked, (C) highest educational attainment and (D) household income level. The association estimates transformed onto the same scale as the Mendelian randomisation estimates are presented in rows prefixed ‘assoc. with depression’, for example, ‘assoc. with depression, prev=0.1’, where prev=0.1 indicates a baseline depression prevalence of 10%. RAPS, Robust Adjusted Profile Score.

Two sample MR estimates of the causal effect of depression liability on outcomes are presented (1) in table 3 for the estimator selected via the Rücker model selection framework, (2) in figures 2 and 3 as forest plots for a subset of estimators, (3) in online supplemental figures S3–S5 as forest plots for all estimators and (4) in online supplemental tables S3–S5 for all estimators.

**Table 3:** Mendelian randomisation causal effect estimates for depression on employment outcomes

Outcome	Method	OR	95% CI	P value
Not in paid employment	IVW fixed effects	1.158	1.064 to 1.26	6.7E-04
Sick/disabled	IVW fixed effects	1.563	1.344 to 1.819	7.4E-09
Caring for home/family	IVW fixed effects	1.164	0.977 to 1.386	9.0E-02
Retired	IVW fixed effects	1.078	0.958 to 1.212	2.1E-01
Unemployed	IVW fixed effects	1.031	0.840 to 1.266	7.7E-01
**Outcome**	**Method**	**Beta**	**95%** **CI**	**P value**
Townsend Deprivation Index	IVW random effects	0.095	−0.0744 to 0.264	2.7E-01
Hours worked	IVW random effects	−0.130	−0.685 to 0.424	6.5E-01
**Outcome**	**Method**	**OR**	**95%** **CI**	**P value**
Highest educational attainment	IVW random effects	1.092	0.938 to 1.273	2.6E-01
Household income	Egger random effects	1.239	0.817 to 1.881	3.2E-01

All outcome effects were adjusted for age, sex, study assessment centre, and genetic principal components. Household income level effect was additionally adjusted for number in household winsorised to 12. We report the estimate of the MR method selected via the Rücker Model selection framework.

The MR estimates can be directly compared with the transformed association study results (table 2 columns suffixed ‘(MR scale)’ and rows prefixed ‘assoc. with depression’ figures 2 and 3). For several outcomes (not in paid employment, unemployment, TDI, highest educational attainment and household income), the transformed association appeared inconsistent with the Rucker selected MR estimate. This suggests that the association study result was confounded for these outcomes.

The MR estimates indicate that depression liability increased the risk of not being in paid employment (OR 1.16, 95% CI 1.06 to 1.26), and this was attributed to it causing increased risk of sickness/disability (OR 1.56, 95% CI 1.34 to 1.82). The MR analyses provided little evidence (p value >0.05) for an effect on caring for home/family (OR 1.16, 95% CI 0.97 to 1.34), early retirement (OR 1.07, 95% CI 0.96 to 1.21), unemployment (OR 1.03, 95% CI 0.84 to 1.27), hours worked (OR −0.13, 95% CI −0.69 to 0.42), TDI (OR 0.10, 95% CI −0.07 to 0.26), household income (OR 1.24, 95% CI 0.82 to 1.88) or highest educational attainment (OR 1.09, 95% CI 0.94 to 1.27). There was little evidence that depression liability effects differed by sex for any outcomes (online supplemental table S9). Full results are presented in online supplemental section 3.

The robustness of MR estimates was investigated. For each outcome, $I_{GX}^{2}$ was around 0.97 (caution advised if <0.9),^23^ and so the degree of NOME assumption violation was minor. Heterogeneity tests suggested effect size heterogeneity across SNPs for hours worked, TDI, household income and highest educational attainment (online supplemental table S6). Consequently, fixed-effect estimates and the maximum likelihood estimate may not be reliable for these outcomes. There was little evidence of unbalanced pleiotropy for any outcome (online supplemental table S7). The Rücker model selection framework results coincided with the heterogeneity test results but not with those of the unbalanced pleiotropy tests, in that Egger regression was selected for highest educational attainment (online supplemental table S8, figures S8 and S12). This did not affect the inferences one would draw. Single SNP MR analyses and leave one SNP out MR analyses produced estimates with approximately Gaussian distributions for all outcomes except caring for home/family (eg, online supplemental figures S6 and S10). Thus, single SNPs are unlikely to be driving results except for this outcome. Also, no overly influential SNPs were identified (using Cook’s distance) for any outcome (eg, online supplemental figures S7 and S11). Robustness analyses are detailed in online supplemental section 3.

## Discussion

We investigated the causal effect of depression liability on employment-related outcomes in the UK Biobank study by using a set of genetic variants robustly associated with depression. Multivariable adjusted regression analyses indicated that people who had suffered depression were more likely to be in non-employment (for any reason) and had greater socioeconomic disadvantage. In contrast, MR analyses suggested that depression liability increased risk of not being in paid employment due to sickness or disability. The MR analyses provided little support for an effect on any other investigated outcome. For about half the outcomes, the regression and MR estimates differed (see figures 2 and 3), suggesting confounding of regression estimates. There was little evidence for depression liability effects on outcomes differing by gender.

The relative importance of health selection and social causation has been investigated with longitudinal observational studies in a range of high-income countries, tending to find stronger evidence for social causation than for health selection.^4 5 26 27^ The strongest evidence for health selection relates to the transition from adolescence to early adulthood (ie, lower educational attainment transitioning into lower status adult occupations),^3 26 28–30^ although a meta-analysis of mental health effects on being employed suggested relatively small effects on transitions from schooling to adult employment.^4^ Our findings did not support an effect of depression liability on education. Regarding health selection operating in later adulthood, several studies show little to no effect,^3 26 31^ but others show effects of adult depression or psychiatric distress on employment and related outcomes (eg, employability, promotion and contract permanence).^3–5 32–34^ Our findings support the health selection hypothesis as we identified effects of depression liability on employment. Our study design did not investigate the social causation hypothesis. Social causation could be occurring alongside health selection. Our analyses were not intended to directly support or refute social causation.

Studies making similar assumptions may erroneously support a biased effect estimate. Using different approaches based on different assumptions and biases (triangulation) can increase confidence in findings or highlight assumptions requiring further examination.^35^ Our study adds to the mixed evidence for effects of depression on socioeconomic disadvantage, giving greater clarity on mechanisms by indicating that depression increases the likelihood of being out of work due to sickness or disability.

Genetic variants are randomly allocated at conception. Consequently, MR is less subject to reverse causation and does not assume no unmeasured exposure–outcome confounding. This is the first study, to our knowledge, to use MR to investigate the effects of depression on reasons for not working. A previous MR study looked at the effect of risk factors (including depression) on social and socioeconomic outcomes in UK Biobank.^36^ In contrast to our study, they did not find evidence for depression being causal on employment outcomes examined, and they found evidence of depression being causal on reduced household income. Their coding for depression was similar to ours but only covered 10 of the 22 UK Biobank assessment centres; their depression associating SNP set was smaller; and they used a PRS as their instrument, rather than using their SNPs as a set of instruments. This may account for result differences between the two studies. Our findings need to be considered in light of potential limitations. There is a well-known selection bias in the UK Biobank cohort, participants tending to be healthier, wealthier and better educated than the general UK population, consistent with a ‘healthy volunteer’ effect.^37^ This could result in our findings not generalising. However, UK Biobank risk factor–trait associations have been found to be similar to the UK population when there is reasonable risk factor variation.^38^ Participant self-selection may have induced collider bias in our findings.^39^ It has been found that genetic variants are not generally correlated with a broad range of 96 behavioural, socioeconomic and physiological baseline factors and so would be unlikely to be subject to strong selection biases.^40^ An unfortunate limitation of our study is that it depends heavily on Caucasian genetic data to address a problem that disproportionately affects ethnic minorities. Restricting our analyses to white British participants aged 40–65 means results may not generalise to other ethnicities or early working-age people. While avoiding standard sources of unmeasured confounding, MR is potentially susceptible to confounding by population structure. We minimised this by restricting the sample to those of white British ancestry and adjusting for GPCs plus assessment centre. A similar covariates set has been shown to adequately account for confounding by birth location for some but not all traits.^41^ Thus, our estimates are as rigorous as we could provide, but some confounding may remain. This is further discussed in online supplemental section 4.^42 43^Another potential source of bias is dynastic effects, that is, parents with high depression genetic propensity, conferring some unrelated advantage/disadvantage regarding employment to their offspring. Sibling MR analysis, which could address this, would be underpowered in the UK Biobank dataset. Our results refer to the UK labour market, specifically that experienced by an older cohort who have lived through weakening of traditional gender roles, and may not apply to markedly different labour market contexts.

The MR estimates we report are average effects on those from age 40 to 65. It could be that the social mechanisms underlying the effect differs across the age groups studied. Similarly, the effects being estimated may differ for younger age groups (for whom we do not have data) and would likely differ in different social contexts, such as in other countries where different social and cultural norms around paid employment operate. Also, UK Biobank subjects were assessed during a relatively short time frame of 5 years, and age at assessment may correlate with temporal changes in society. Fortunately, age is a covariate in all the regressions, so adjustment has been made for this.

Regarding our MR, we have referred throughout to the effect of depression liability on outcome. When interpreting the MR results, estimates reflect the effect of liability to depression on the outcome. Some people will experience a degree of this latent liability to depression without ever actually experiencing depression, and this liability may affect their outcomes. Therefore, our instrument SNPs do not solely instrument the binary exposure ‘having depression’ but rather the effect of an underlying liability to depression.^44^

## Conclusions

The effect estimates for depression liability on sickness and disability are important, considering the high societal and personal costs of inability to work for health reasons. Depression has high prevalence and can have strong adverse effects on day-to-day functioning for extended periods of time. Consequently, the global burden it imposes is one of the highest for any disorder.^45^ In Britain, the proportion of sickness and disability-related benefits claimants accounted for by depressive disorders rose from 8.9% in 1995 to 20.6% in 2014 despite little change in claimant numbers.^46^ Societal costs include workforce absenteeism and presenteeism, suicidality and comorbidities, and their societal consequences. Depression treatment has been well researched, and effective treatments are available.^47^ Despite this, depression treatment is underfunded worldwide. Return on investment in increased depression treatment has been estimated at approximately 2 to 1 for healthcare and 3 to 1 if indirect costs are included.^48^

Our finding that depression liability increased sickness/disability risk raises the potential that early intervention through effective healthcare could mitigate the adverse societal impacts of depression by increasing working capacity within the population. It also may inform the development of workplace interventions tailored for individuals with mental health issues, such as individual placement and support.^49^ Further research is advisable to explore whether this is so and to quantify impact. Evaluations of mental health interventions or policies should consider collecting information on employment-related outcomes, especially being out of work due to sickness/disability.

**What is already known on this subject**

- The relative importance of depression being causal on socioeconomic disadvantage (health selection) and the reverse causal direction (social causation) haves been investigated with longitudinal observational studies in a range of high-income countries.
- These have tended to find stronger evidence for social causation than for health selection, for which the evidence is mixed.

**What this study adds**

- We used Mendelian randomisation (a study design different from those used in previous studies) to investigate the health selection hypothesis, specifically, the effect of depression liability on different reasons for not working and on income, education, deprivation and hours worked.
- Our findings, relating to working-age people over 40, support an effect of depression on employment (through increased sickness and disability) but not on the other socioeconomic indicators examined.
- This may justify government intervention, as improving people’s mental health has potential to reduce adverse employment effects.

## Data Availability

Data may be obtained from a third party and are not publicly available. Access to the dataset upon which this study is based must be sought from UK Biobank. The code used in the study analyses is available upon request from the authors.
