# Supplementary material for: Effects of depression on employment and social outcomes: a Mendelian randomisation study
Source: J Epidemiol Community Health. 2022 Mar 22;76(6):563–71. doi: 10.1136/jech-2021-218074 (PMC9118074; doi:10.1136/jech-2021-218074)
Supplement: Supplementary data [file jech-2021-218074supp001.pdf]

Appendix: Effects of depression on employment status: A Mendelian randomisation study

1 Purpose

This is the appendix for the paper

- Effects of depression on employment status: A Mendelian randomisation study

It provides further details to the paper and describes supplementary files.

Table of Contents

1 Purpose 1
2 Methods 2
2.1 UK Biobank fields 2
2.2 Study population 2
2.3 Depression SNPs 2
2.4 Polygenic score 3
2.5 Outcomes 3
2.6 Exposure 3
2.7 Regression of Outcomes on Exposure 3
2.8 Regression of Exposure on Polygenic score 4
2.9 SNP Associations with Outcomes 4
2.10 MR Analyses 5
2.11 Investigation of interaction with sex 5
2.12 Outlier SNP removal 6
3 Results 6
3.1 Result Files 6
3.2 Regression of Outcomes on Exposure 7
3.3 Regression of Exposure on Polygenic score 7
3.4 MR Analyses 7
3.5 MR Robustness analyses 8
3.5.1 Sick/Disabled 9
3.5.2 Not in Paid employment 9

Campbell D, *et al. J Epidemiol Community Health* 2022; 76:563–571. doi: 10.1136/jech-2021-218074

3.6 Sex Stratified Analyses 10
4 Discussion 10
5 Figures 11
6 Tables 19
7 References 29

2 Methods

2.1 UK Biobank fields

A list of the important UK Biobank fields used in this study is given in Table S10.

2.2 Study population

UK Biobank participants were excluded for the following reasons

- Not white UK (UK Biobank field 22006)
- Over retirement age at time of assessment (60 years female, 65 years male)
- Participant did not have a value for any of the outcomes of interest.
- mismatch between self-declared sex and sex genetically predicted (UK Biobank field 22001)
- abnormal number of X and Y chromosomes (UK biobank field 22019)
- Low genotyping rate (<98.5%), which is indicative of low-quality DNA (UK Biobank field 22005)
- Participant DNA is an outlier for heterozygosity or missing rate, which implies that the genotypes for these samples are of poor quality (UK Biobank field 22027)
- Participant withdrawn from the study (before 4th February 2020)
- Over-relatedness: if a pair surviving the above exclusions were too genetically related (kinship > 0.042, e.g. closer than 2nd cousins), then one of the pair was dropped. The participant with the most relations was preferentially dropped. If both participants had the same number of relations, then the subject dropped was randomly chosen.

A STROBE flowchart (Figure S1) reports the numbers lost through these exclusion criteria. The retained sample (230,790 participants) was the basis of all further analysis.

2.3 Depression SNPs

We constructed a genetic instrument to investigate the effect of depression on occupation outcomes. A recent study reported on Single Nucleotide Polymorphisms (SNPs) associating with depression.[1] From the authors Howard et al., we obtained their association results after exclusion of their UK Biobank cohort, i.e. based on just their PGC and 23andMe cohorts. SNP associations not reaching genome-wide significance (p-value = 5×10^{-8}) were discarded. The following SNP exclusion criteria were then applied

Campbell D, et al. J Epidemiol Community Health 2022; 76:563–571. doi: 10.1136/jech-2021-218074

- Hardy Weinberg Equilibrium (HWE): We used Family Wide Error Rate (FWER) = 1 Bonferroni correction to screen for SNPs not in HWE. In other words, we would expect one perfectly good SNP to be rejected according to this criterion.
- Low information content: Information score ranges from 0 to 1 and reflects the quality of sequencing. SNP dropped if < 0.9
- Low Minor Allele Frequency (MAF): SNP dropped if MAF < 0.01
- Palindromic and high MAF: Palindromic SNP dropped if MAF > 0.4

The last criterion arises from the difficulty of reconciling the strands palindromic SNPs were called on, in the SNP-exposure and SNP-outcome datasets. Linkage Disequilibrium (LD) clumping of the remaining SNP set was used to identify a set of 30 mutually independent SNPs. This was done using the ieugwasr R package ld_clump function with default clumping window size of 10 MBases and a cut-off of R2 < 0.01. These SNPs (listed in Table S11) constituted our instrument SNP set used for subsequent Mendelian Randomisation analyses. A flow chart for the generation of the instrument SNP set is shown in Figure S2. Associations were harmonised (using the TwoSampleMR R package) to ensure consistent directions of association across all SNPs.

2.4 Polygenic score

In order to measure the strength of our genetic instruments and to validate the instrument-exposure association assumption of the Mendelian randomisation method, we generated a polygenic score for the exposure for each participant. This was constructed as the number of risk alleles the subject carried for a SNP, summed over all instrument SNPs. Scores were generated using the score function of PLINK 1.9.[2] We applied the default settings which impute any missing genotype with its expectation, (i.e. twice the SNP's risk allele frequency).

2.5 Outcomes

The main paper Methods, Outcomes section covers this adequately.

2.6 Exposure

A dichotomous indicator variable for depression was created by combining self-report and Hospital Episode Statistics variables available on UK Biobank subjects. Depression was indicated by depression diagnosis, either a hospital inpatient ICD 9 (matching 296* or 311*) or ICD 10 (matching F32* or F23*) code, self-reported depression, or self-report of seeing a psychiatrist for depression, anxiety or tension. Details are indicated in Table S10. Prevalence of this depression phenotype in UK Biobank subjects was 12% and 15.9% in males and females respectively. This phenotype was used for association analyses and polygenic risk score regression. The MR analyses estimates relate to the Howard et al. depression phenotype.

2.7 Regression of Outcomes on Exposure

We regressed the outcomes on the exposure using the following regression equation.

$$Outcome = Exposure + sex + age + assessmentCentre + GPC1 + GPC2 + \cdots + GPC40$$

Where

- Exposure = depression
- Sex – coded as male =1, female =2

Campbell D, et al. J Epidemiol Community Health 2022; 76:563–571. doi: 10.1136/jech-2021-218074

- AssessmentCentre – the UK Biobank assessment centre. These were represented in the regression by a set of dichotomous dummy variables.
- GPC1 … GPC40 – genetic principal components

For ordinal, binary and continuous outcomes we used ordinal, logistic and linear regression respectively. The logistic and linear regressions were implemented using PLINK.[2] The ordinal regressions were implemented using the *polr* function of the MASS R package. For some categories of the non-continuous outcomes, the count was low or even zero for some assessment centres. This would cause the regression to fail or return inaccurate estimates for these assessment centres. To overcome this, assessment centres were repeatedly merged until the minimum such count exceeded 50 prior to performing regressions. In all of the outcomes, at most three assessment centres were merged in this way.

To facilitate comparison of the MR and association study estimates, the estimate for the regression of each outcome on depression was transformed onto the same scale as the MR estimates. The MR estimate is the SNP outcome association (pre link function) divided by the SNP exposure association (pre link function). By pre link function association we mean the regression coefficient from the linear predictor of the generalised linear model. For the exposure of interest (dichotomous depression) logistic regression yields a SNP exposure association (pre link function) that is the predicted change in a latent standard logistic distributed liability for the exposure. The difference between the average exposure liability of those affected and those unaffected can be calculated. The association study estimate is transformed onto the same scale as the MR estimate by dividing the (pre link function) association study estimate by this mean difference. A parameter of this transformation is the exposure lifetime prevalence. In our case the exposure was the Howard et al. trait, a combination of (i) in the PGC cohort - clinically diagnosed Major Depression Disorder (MDD), and (ii) in the 23andMe cohort - self-declared clinical depression. Diagnostic and Statistical Manual of Mental Disorders (DSM-IV) MDD has a lifetime prevalence of around 15%, and women are at twice the risk as men.[3] Therefore, depression prevalences of 10% and 20% are appropriate for males and females respectively. These prevalences were used in transforming the association results onto the MR scale.

2.8 Regression of Exposure on Polygenic score

We regressed exposure on the polygenic score for the exposure, using logistic regression. The regression equation was

$$Exposure = PolygenicScore + sex + age + assessmentCentre + GPC1 + GPC2 + \cdots + GPC40$$

We conducted ANOVA and calculated the relative likelihood for each regressor. The relative likelihood is the p-value for the null hypothesis that the regression without the regressor is just as good a fit as the regression with the regressor.

2.9 SNP Associations with Outcomes

We regressed each outcome on each SNP of the instrument set using the UK Biobank dataset. The regression equation was

$$Outcome = riskAlleleCount + sex + age + assessmentCentre + GPC1 + GPC2 + \cdots + GPC40$$

Where

Campbell D, *et al. J Epidemiol Community Health* 2022; 76:563–571. doi: 10.1136/jech-2021-218074

- $riskAlleleCount$ – the risk allele count (0, 1 or 2) for the SNP in question

We used ordinal, logistic and linear regression for ordinal, dichotomous and continuous outcomes respectively. The logistic and linear regressions were implemented using PLINK.[2] The ordinal regressions were implemented using the polr function of the MASS R package. For some categories of the non-continuous outcomes, the count was low or even zero for some assessment centres. This would cause the regression to fail or return inaccurate estimates for these assessment centres. To overcome this, assessment centres were repeatedly merged until the minimum such count exceeded 10 prior to performing regressions. In all of the outcomes, at most three assessment centres were merged in this way.

2.10 MR Analyses

We conducted a two-sample MR analysis using the SNP-exposure associations obtained from Howard *et al.* and the SNP-outcome associations from the study sample. We estimated causal effects using the wide range of MR causal effect estimation methods made available by the TwoSampleMR R package.[4] We also tried using methods from the RadialMR R package. The Radial MR-Egger method is a regression directly on a Galbraith radial plot. However, for some outcomes the Radial MR-Egger fit as displayed on the (RadialMR generated) plot was clearly wrong. Also, for several outcomes the Radial MR-Egger estimate was very different from all the other estimates. In addition, we could not update the package from its GitHub repository. So we have excluded RadialMR package estimators from our reporting.

A sensitivity analysis was conducted on the MR analyses results. Heterogeneity amongst the causal effect estimates from the instrument SNPs was assessed with Cochran's Q (assuming balanced pleiotropy) and Rücker's Q (assuming unbalanced pleiotropy). Using Cochran's Q and Rücker's Q as inputs, we applied the Rücker model selection framework to identify the best fitting model between fixed effect and random effect versions of the IVW and Egger methods.[5] We followed Bowden et al. in using 0.05 as a significance threshold for detecting pleiotropy for model selection purposes (see Box 3 of Bowden et al. 2018).[6] We conducted unbalanced pleiotropy tests (implemented via TwoSampleMR::mr_pleiotropy_test). We also calculated I^2_{GX}, a measure of the degree of violation of the No Measurement Error (NOME) assumption for SNP-exposure associations (implemented via TwoSampleMR::Isq). We conducted one SNP MR analyses and Leave One SNP Out MR analyses.

2.11 Investigation of interaction with sex

Given the well-established differences in employment by sex, we conducted sex-stratified MR analyses. We tested whether there was evidence for difference in causal effects between the sexes for each outcome. We did this using Fisher's z-score method (a Wald test), comparing z to a standard normal distribution, by applying:

$$z = \frac{b_{Male} - b_{Female}}{\sqrt{se^2_{Male} + se^2_{Female}}}$$

Where

- b = causal effect estimate
  - for dichotomous outcomes = log odds ratio
  - for continuous outcomes = beta (regression coefficient)
- se = standard error of b

- subscript denotes stratum

The Rücker model selection framework was used to identify which causal effect estimate to use per stratum outcome combination. We used the same instrument SNP set for the sex stratified MR analyses as for the main MR analyses. As the Howard et al. exposure SNP association estimates were from a discovery dataset these associations are likely to be regressed towards the mean in the UK Biobank dataset. This could bias evidence for sex difference in causal effect if the degree of regression towards the mean differed between sexes. However, there is no reason to expect this given the Howard et al. dataset and the UK Biobank dataset male to female ratios are similar.

2.12 Outlier SNP removal

As a further sensitivity analysis, we attempted to identify and exclude from the instrument SNP set, SNPs that were overly influential on MR analyses results. SNP influence was measured using the SNP's Cook's distance from the Egger regression fit. We used the median point of the F distribution F(p=0.5, 2, #snps-2) as a threshold for outlier detection. Our intension was to repeat our MR analyses using a reduced SNP instrument set from which overly influential SNPs had been excluded. However no overly influential SNPs were detected.

3 Results

3.1 Result Files

Result files are available upon request from the authors as a zip file. Result files referred to in this document normally have names in the following form, or some subset thereof

- <<PREFIX>>_<<SUBJECTS>>_<<EXPOSURE>>_<<OUTCOME>>_<<OUTLIER>>*

Where

<<SUBJECTS>> indicates the subject dataset, and is one of

- ukb_17333_dep_H23amPgc.subjects.qced – qc'ed UK Biobank subjects
- ukb_17333_dep_H23amPgc.subjects.qced.Male – Male qc'ed UK Biobank subjects
- ukb_17333_dep_H23amPgc.subjects.qced.Female – Male qc'ed UK Biobank subjects

<<EXPOSURE>> is

- bDepression = depression

<<OUTCOME>> indicates the employment outcome, and is one of

- iSickNotEmp - indicates the Sick/disabled outcome
- iFamilyNotEmp - indicates the Caring for Home/Family outcome
- iRetiredNotEmp - indicates the Early Retirement outcome
- iUnempNotEmp - indicates the Unemployed outcome
- iOtherNotEmp - indicates the Not in Paid Employment outcome
- f.767.0.0 - indicates the Work Hours Weekly outcome
- f.189.0.0 - indicates the deprivation (TDI) outcome

<<OUTLIER>> indicates the instrument SNP set, and is one of

- incOutlier – results for the full SNP instrument set
- excOutlier – results for the reduced SNP instrument set (excluding outlier SNPs). No such files are presented

3.2 Regression of Outcomes on Exposure

Table S1 reports on the regressions of outcomes on depression. For each outcome the five most statistically significant regressors are reported.

These results are also to be found in files

- collateRegressOutcomeOnExposure_ukb_17333_dep_H23amPgc.subjects.qced_ageSexCentreGpc_OutcomeRegressor.csv
- collateRegressOutcomeOnExposureLinear_ukb_17333_dep_H23amPgc.subjects.qced_ageSexCentreGpc_OutcomeRegressor.csv
- collateRegressOutcomeOnExposureOrdinal_ukb_17333_dep_H23amPgc.subjects.qced_ageSexCentreGpc_Regressor.csv
- collateRegressOutcomeOnExposureOrdinal_ukb_17333_dep_H23amPgc.subjects.qced_ageSexNinHouseCentreGpc_Regressor.csv

3.3 Regression of Exposure on Polygenic score

The regression of depression on polygenic score is presented in Table S2.

These results are also found in file

- regressExposureOnPolygenicScore_ukb_17333_dep_H23amPgc.subjects.qced_ageSexCentreGpc_bDepression.csv

3.4 MR Analyses

The two sample MR causal effect estimates for all the outcomes are presented in Table S3, Table S4 and Table S5. They results are also presented graphically in forest plots in Figure S3, Figure S4 and Figure S5. Results for the 'sign concordance test' method are not presented as this method did not return any precision estimate. Results for the 'Unweighted regression' method are not presented as this method reported huge (probably wrong) confidence intervals.

The tables are also available in files

- collateMrAnalyses_ukb_17333_dep_H23amPgc.subjects.qced_bDepression_<<A>>_<<B>>_AllCausalEffectEstimates_incOutlier.csv
- collateMrAnalysesBeta_ukb_17333_dep_H23amPgc.subjects.qced_bDepression_<<A>>_<<B>>_AllCausalEffectEstimates_incOutlier.csv

The forest plots are available in files

- collateMrAnalyses_ukb_17333_dep_H23amPgc.subjects.qced_bDepression_<<A>>_<<B>>_forestPlot_allMethods. pdf
- collateMrAnalysesBeta_ukb_17333_dep_H23amPgc.subjects.qced_bDepression_<<A>>_<<B>>_forestPlot_allMethods.pdf

Campbell D, et al. J Epidemiol Community Health 2022; 76:563–571. doi: 10.1136/jech-2021-218074

where

- <<A>> is a label for a set of outcomes
- <<B>> is a label for the set of covariates adjected for

3.5 MR Robustness analyses

Robustness of the MR analyses results was investigated in several ways.

Heterogeneity test results are presented in Table S6.

Unbalanced pleiotropy test results are presented in Table S7.

Rücker model selection framework results are presented in Table S8.

We also intended to repeat the full MR analyses plus sensitivity analysis on a reduced instrument SNP set from which overly influential SNPs had been excluded. However no overly influential SNPs were detected for any outcome.

These results are also available in files

- collateMrAnalyses_ukb_17333_dep_H23amPgc.subjects.qced_bDepression_<<A>>_<<B>>_<<C>>_<<D>>. csv
- collateMrAnalysesBeta_ukb_17333_dep_H23amPgc.subjects.qced_bDepression_<<A>>_<<B>>_<<C>>_<<D>>. csv

where

- <<A>> (outcome set identifier) = NotEmp | Ordinal | Beta
- <<B>> (covariates id) = ageSexCentreGpc | ageSexNinHouseCentreGpc
- <<C>> (result type) = HeterogeneityTest | BalancedPleiotropyTest | RückerModelSelection | I2gx | AllCausalEffectEstimates | RepresentativeCausalEffectEstimate
- <<D>> (SNP set) = incOutlier | excOutlier

In addition, for each outcome, the following diagnostic plots were generated

- scatterplot of SNP-outcome versus SNP-exposure association
- forest plot of causal effect estimates
- QQ plot of Single SNP causal effect estimates
- QQ plot of Leave One SNP Out causal effect estimates
- Rücker Model Selection Framework plot
- QQ plot of SNP Cochran Q

These plots are available in files

- do2SampleMrAnalyses_ukb_17333_dep_H23amPgc.subjects.qced_bDepression_<<Outcome>>_<<Covariates>>_<< snpSetId >>.pdf

where

Campbell D, et al. J Epidemiol Community Health 2022; 76:563–571. doi: 10.1136/jech-2021-218074

- <<Outcome>> (outcome) = iSickNotEmp | iFamilyNotEmp | iRetiredNotEmp | iUnempNotEmp | iOtherNotEmp | f.767.0.0 (=Hours Worked) | f.189.0.0 (=TDI) | maxEducLevel | householdIncome
- << Covariates >> (covariates id) = ageSexCentreGpc | ageSexNinHouseCentreGpc
- << snpSetId>> (SNP set) = incOutlier | excOutlier

For most outcomes, the leave one SNP out analysis and per SNP analysis gave approximately Gaussian causal effect estimates, barring a few outliers. For most outcomes, the SNP's contribution of Cochran's Q followed a Chi^2 df=1 distribution, barring a few outliers. For those outcomes for which there was heterogeneity in effect size, the SNP's contribution of Cochran's Q appears to follow an inflated Chi^2 distribution.

3.5.1 Sick/Disabled

We present results for a specimen outcome, the Sick/Disabled outcome. A Scatter plot of Sick/Disabled-SNP associations (estimated from our UK Biobank analytical sample) versus exposure-SNP associations (from Howard et al.) are presented in Figure 1 (main text). Quantile-Quantile plots comparing Single SNP and Leave One SNP Out causal effect estimates for depression on the Sick/Disabled outcome against Gaussian distributions, are presented in Figure S6. Figure S7 shows a QQ plot comparing the SNP contributions to Cochran's Q appear to a Chi2 df=1 distribution. The Rücker model selection framework's preferred model (Figure S8).

These plots are taken from file

- do2SampleMrAnalyses_ukb_17333_dep_H23amPgc.subjects.qced_bDepression_iFamilyNotEmp_ageSexCentreGpc.pdf

A similar file is available for each outcome.

3.5.2 Not in Paid employment

We present results for a specimen outcome, the Not in Paid employment outcome. A scatter plot of outcome-SNP associations (estimated from our UK Biobank analytical sample) versus exposure-SNP associations (from Howard et al.) are presented in Figure S9. Quantile-Quantile plots comparing Single SNP and Leave One SNP Out causal effect estimates for depression on the Sick/Disabled outcome against Gaussian distributions, are presented in Figure S10. Figure S11 shows a QQ plot comparing the SNP contributions to Cochran's Q appear to a Chi2 df=1 distribution. The Rücker model selection framework's preferred model (Figure S12).

These plots are taken from file

- do2SampleMrAnalyses_ukb_17333_dep_H23amPgc.subjects.qced_bDepression_iOtherNotEmp_ageSexCentreGpc.pdf

A similar file is available for each outcome.

Campbell D, *et al. J Epidemiol Community Health* 2022; 76:563–571. doi: 10.1136/jech-2021-218074

3.6 Sex Stratified Analyses

MR analyses were repeated in male only and female only subsets of the study sample for all outcomes. A Wald test was used to compare the male only and female only causal effect estimates of depression on each outcome. There was little evidence for differences in causal effect across sex.

The full results for the sex stratified MR analyses are presented in files matching

- *_ukb_17333_dep_H23amPgc.subjects.qced.Male_*
- *_ukb_17333_dep_H23amPgc.subjects.qced.Female_*

The Wald test results are available in files

- collateBetaCausalEffectDiffBetweenStrata_MaleFemale_<<covariates>>_<<snpSet>>*csv

4 Discussion

Here we elaborate on limitations of our study.

Two Sample MR assumes the exposure-SNP and outcome-SNP associations are obtained from independent datasets. In our case exposure-SNP associations were obtained from the Howard et al. dataset after exclusion of the UK Biobank cohort, so this assumption was met.

Ideally in MR analyses, the outcome-SNP and exposure-SNP regressions control for the same covariates. Our outcome SNP regressions controlled for age, sex, genetic principal components and assessment centre. The exposure-SNP regressions adjusted for similar covariates. The exposure-SNP regressions we used were obtained by meta-analysis of association studies from two cohorts (23andMe and PGC). Associations estimated from the 23andMe cohort were adjusted for age, sex, and the top 5 genetic principal components to account for residual population structure.[7] Associations for the PGC cohort were obtained by a meta-analysis of genome wide association studies done in the constituent cohorts. We anticipate most of these will also have adjusted for age, sex and genetic principal components.[8]

The exposure-SNP association inputs are likely to be inflated by winner's curse as we took our exposure-SNP associations from a discovery association study. This inflation would tend to deflate causal effect estimates and their significance. The estimate of I_{GX}^2 at around 0.97 implies the degree of regression dilution would have negligible effect on the magnitude of our casual effect estimates.

For some outcomes individual SNP contributions to Cochran's Q appear distributed as an inflated ChiSq df=1 distribution. This heterogeneity in casual effect estimates across SNPs indicates horizontal pleiotropy and/or un-modelled confounders of the SNP-outcome regression. Some of the estimation methods employed are robust to such heterogeneity, however these estimation methods make the Instrument Strength Independent of Direct Effect (InSIDE) assumption.

Selection bias is a concern for MR study designs. Such selection can induce paths between the casual ancestors of any variable influencing likelihood of study inclusion. If both exposure and outcome were such casual ancestors, then paths would be induced between instrument SNPs and outcome. This would bias the regression of outcome on SNP (via collider bias). Furthermore, the strength of such induced paths, and thence the regression bias, would depend on the SNP exposure association strength, violating the InSIDE assumption.

Campbell D, *et al. J Epidemiol Community Health* 2022; 76:563–571. doi: 10.1136/jech-2021-218074

5 Figures

Figure S1 STROBE flowchart of UK Biobank participant exclusions

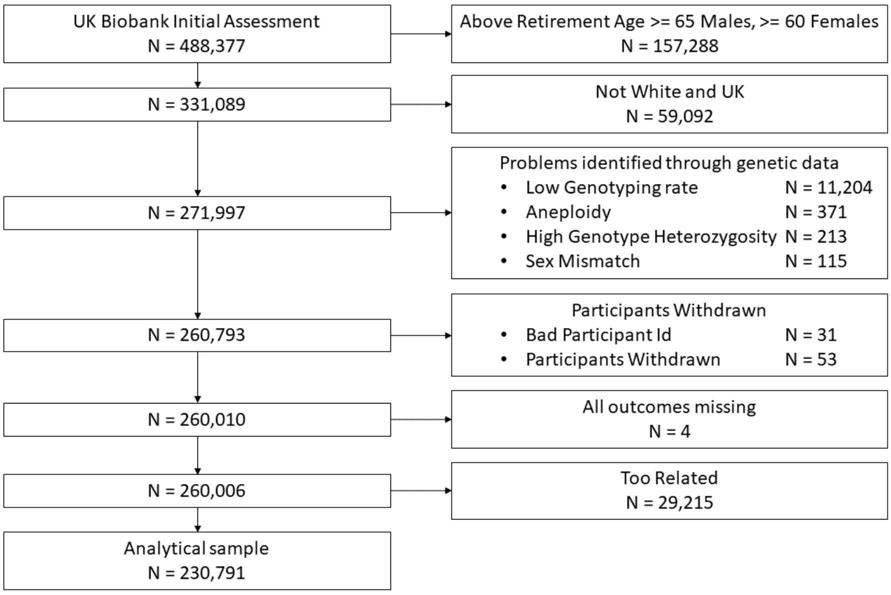

Figure S2 Flowchart of SNP screening for MR instrument

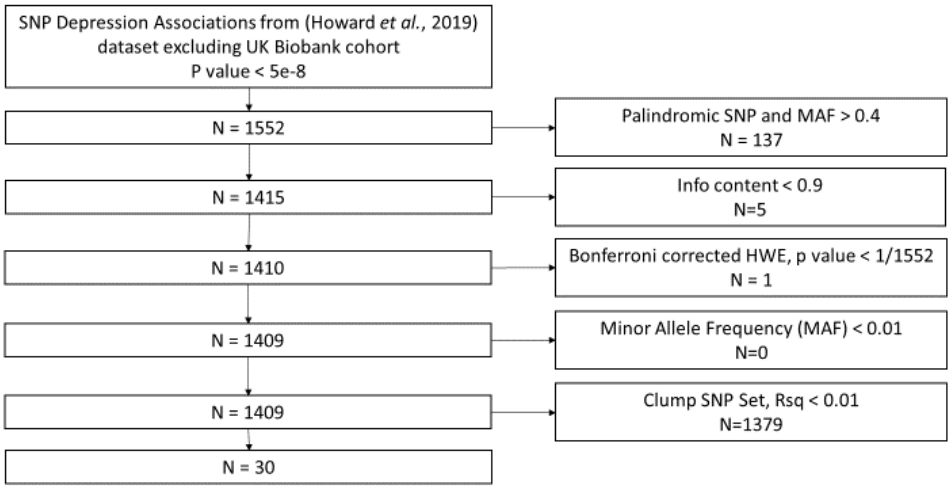

Figure S3 Forest plots of causal effect estimates of depression on employment category outcomes.

Campbell D, *et al. J Epidemiol Community Health* 2022; 76:563–571. doi: 10.1136/jech-2021-218074

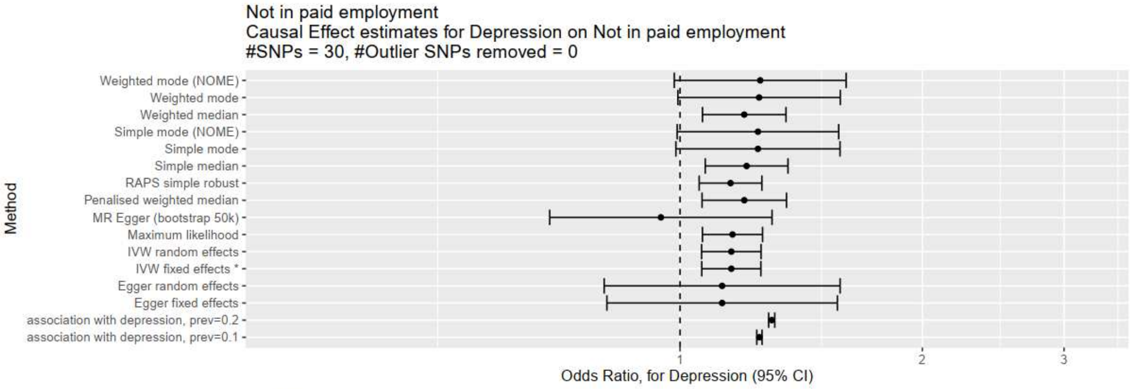

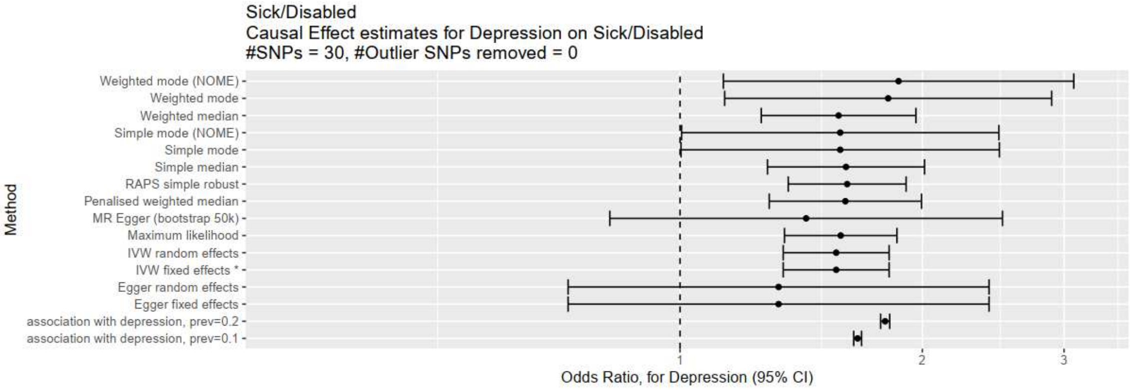

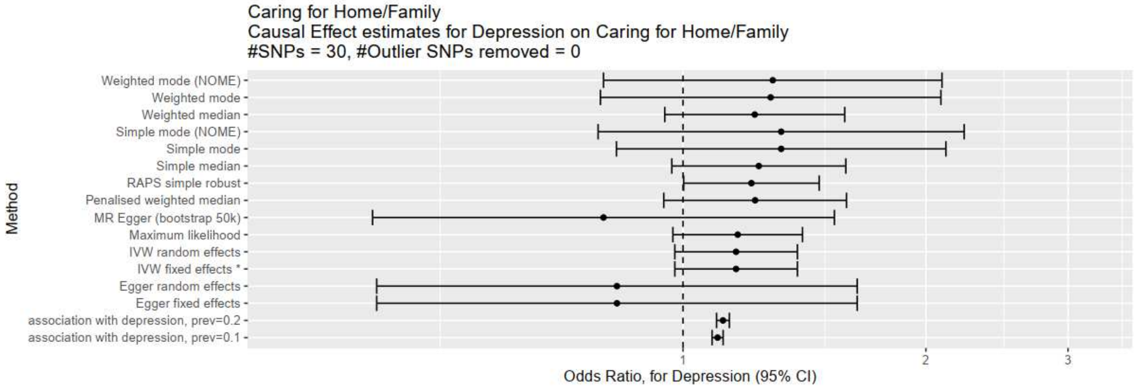

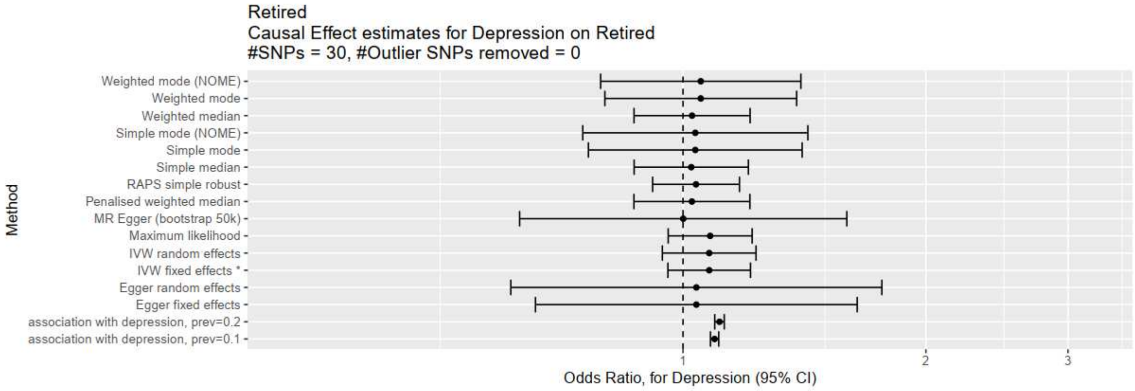

Campbell D, *et al. J Epidemiol Community Health* 2022; 76:563–571. doi: 10.1136/jech-2021-218074

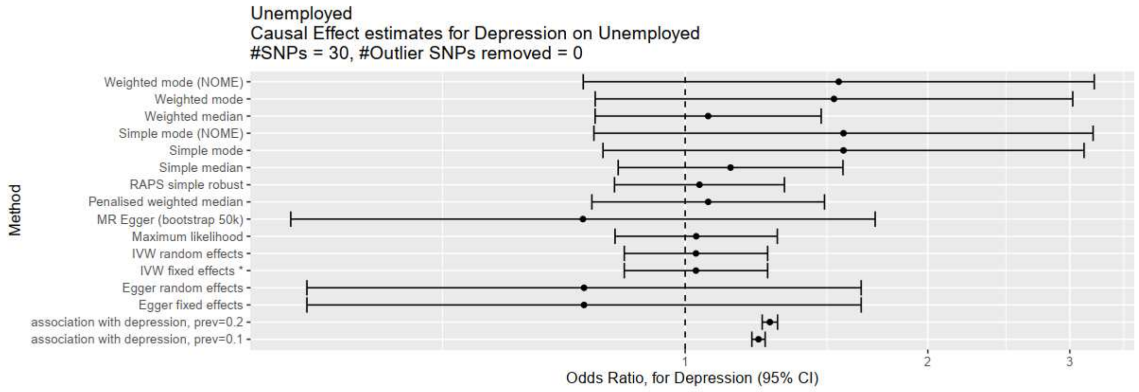

Footnote: MR analyses results for full instrument set. Causal effect estimate (plus 95% Confidence Interval) for change of depression status from unaffected to affected. The association estimates transformed onto the same scale as the MR estimates are presented in rows titled e.g. 'association with depression, prev=0.1', where prev=0.1 indicates a baseline depression prevalence of 10%.

Figure S4 Forest plots of causal effect estimates for depression on Weekly Hours Worked and TDI outcomes

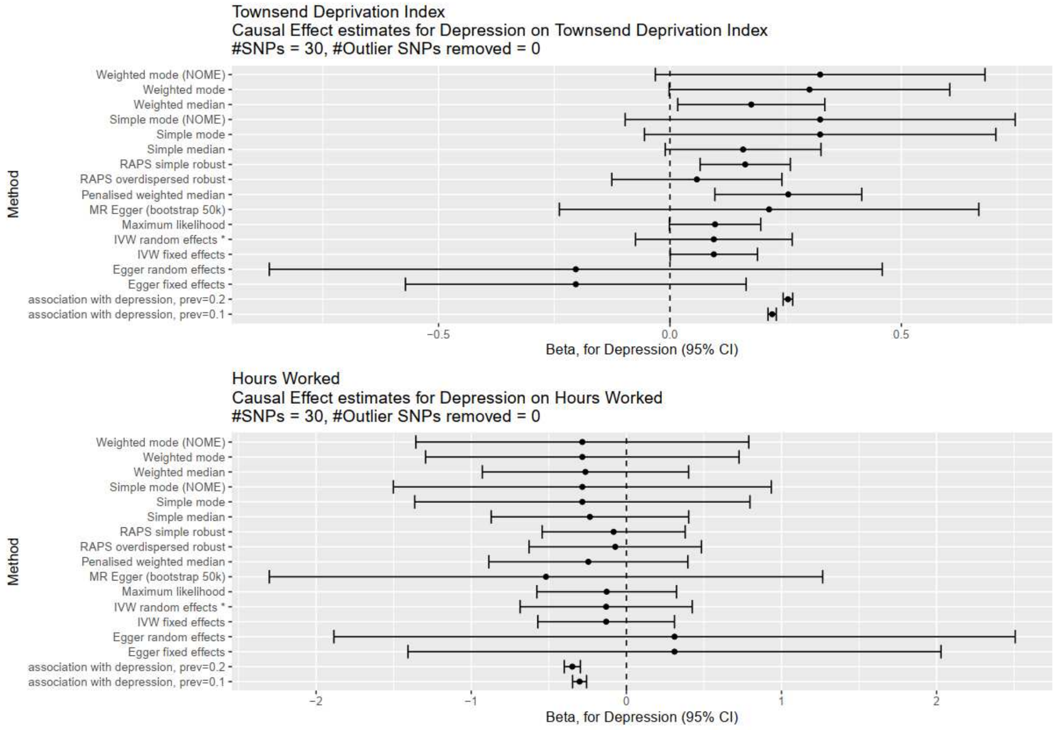

Campbell D, *et al. J Epidemiol Community Health* 2022; 76:563–571. doi: 10.1136/jech-2021-218074

Footnote: MR analyses results for full instrument set. Causal effect estimate (plus 95% Confidence Interval) for change of depression status from unaffected to affected. The association estimates transformed onto the same scale as the MR estimates are presented in rows titled e.g. 'association with depression, prev=0.1', where prev=0.1 indicates a baseline depression prevalence of 10%.

Figure S5 Forest plots of causal effect estimates for depression on Household Income Level and Highest Educational Attainment

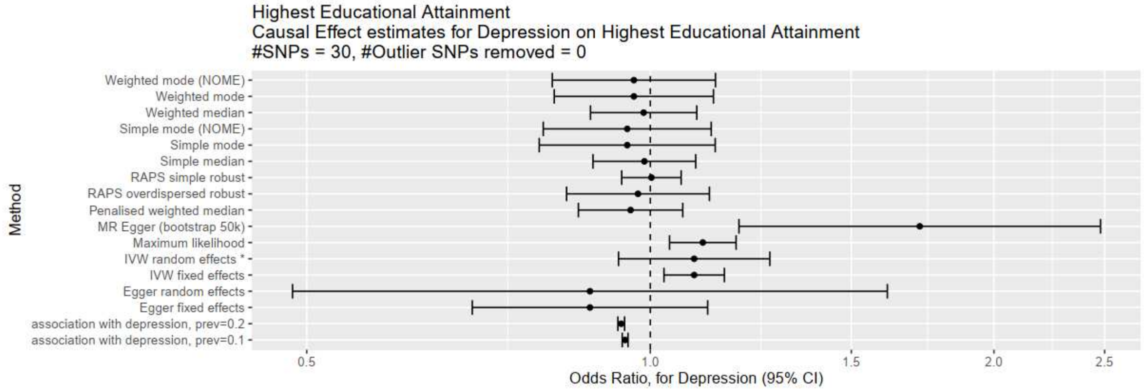

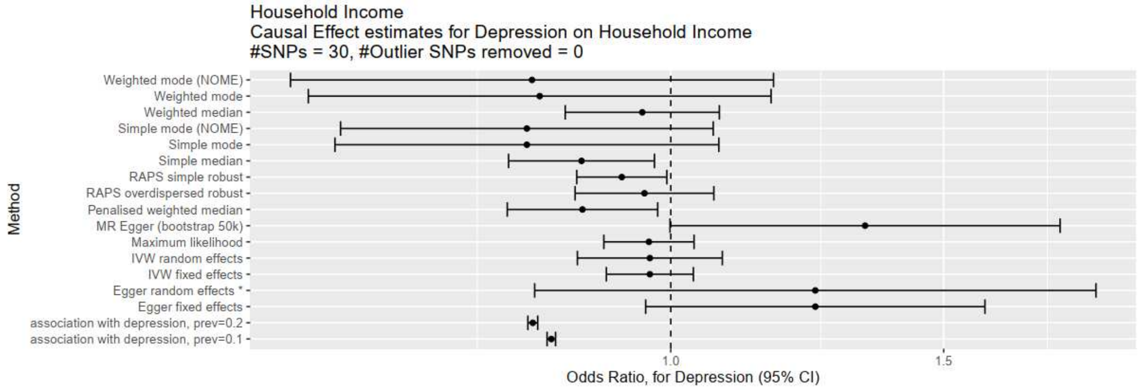

Footnote: MR analyses results for full instrument set. Causal effect estimate (plus 95% Confidence Interval) for change of depression status from unaffected to affected. The association estimates transformed onto the same scale as the MR estimates are presented in rows titled e.g. 'association with depression, prev=0.1', where prev=0.1 indicates a baseline depression prevalence of 10%.

Figure S6 Quantile-Quantile plots comparing Single SNP and Leave One SNP Out causal effect estimates for depression on the Sick/Disabled outcome against Gaussian distributions.

Campbell D, *et al. J Epidemiol Community Health* 2022; 76:563–571. doi: 10.1136/jech-2021-218074

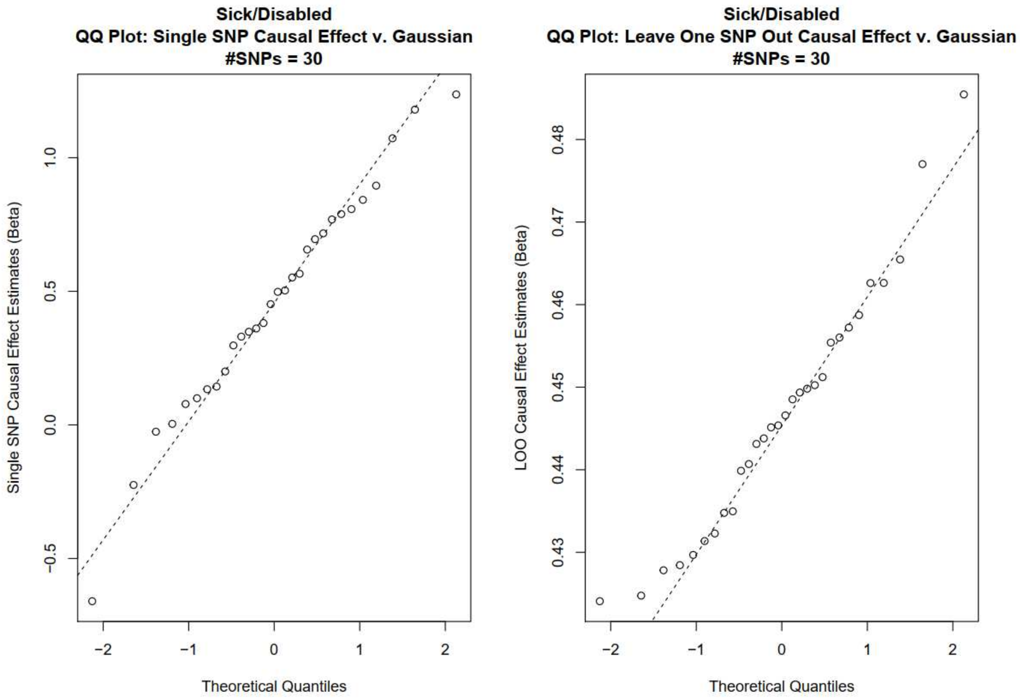

Figure S7 QQ Plots comparing SNP contribution to Cochran's Q to a Chi² df=1 distribution for Sick/Disabled outcome.

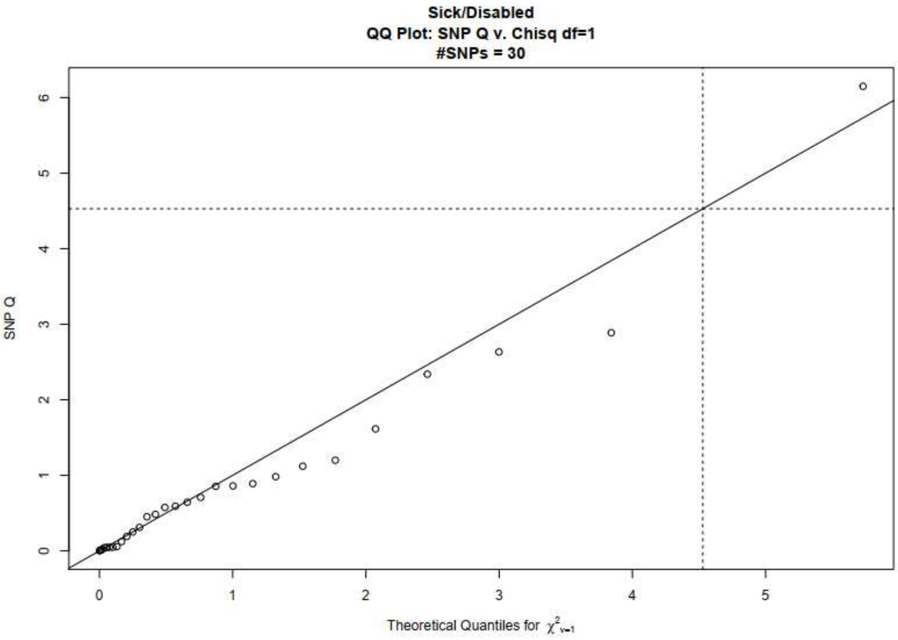

Campbell D, *et al. J Epidemiol Community Health* 2022; 76:563–571. doi: 10.1136/jech-2021-218074

Figure S8 Rücker model Selection Framework Plots for Sick/Disabled outcome.

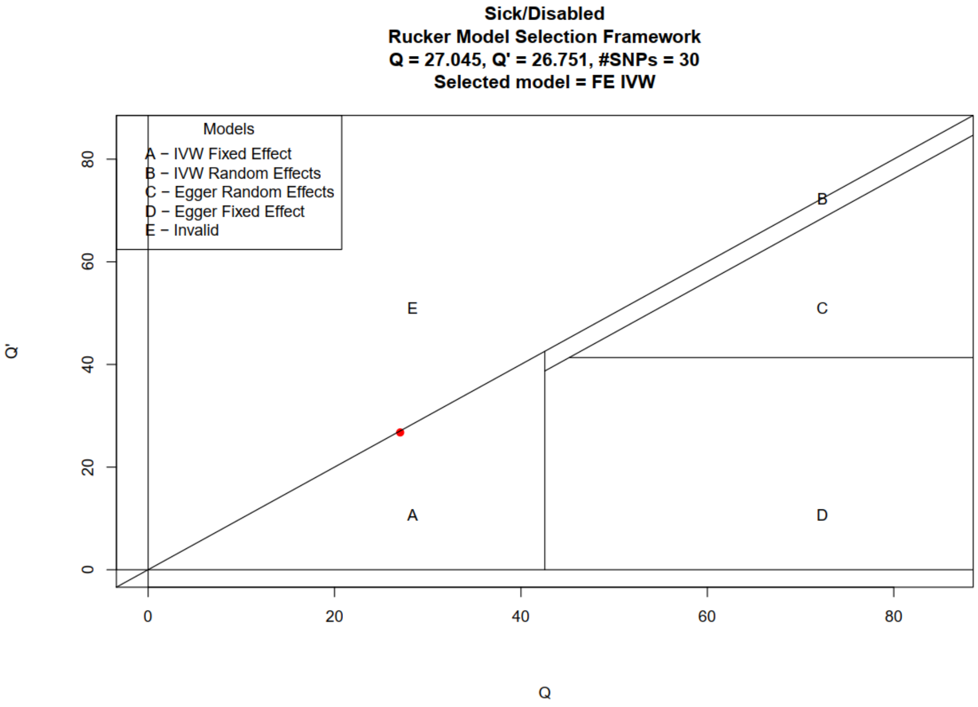

Footnote: The region in which the red dot lies indicates the model selected.

Figure S9 Scatter plot of Not in paid employment-SNP associations versus exposure-SNP associations

Campbell D, *et al. J Epidemiol Community Health* 2022; 76:563–571. doi: 10.1136/jech-2021-218074

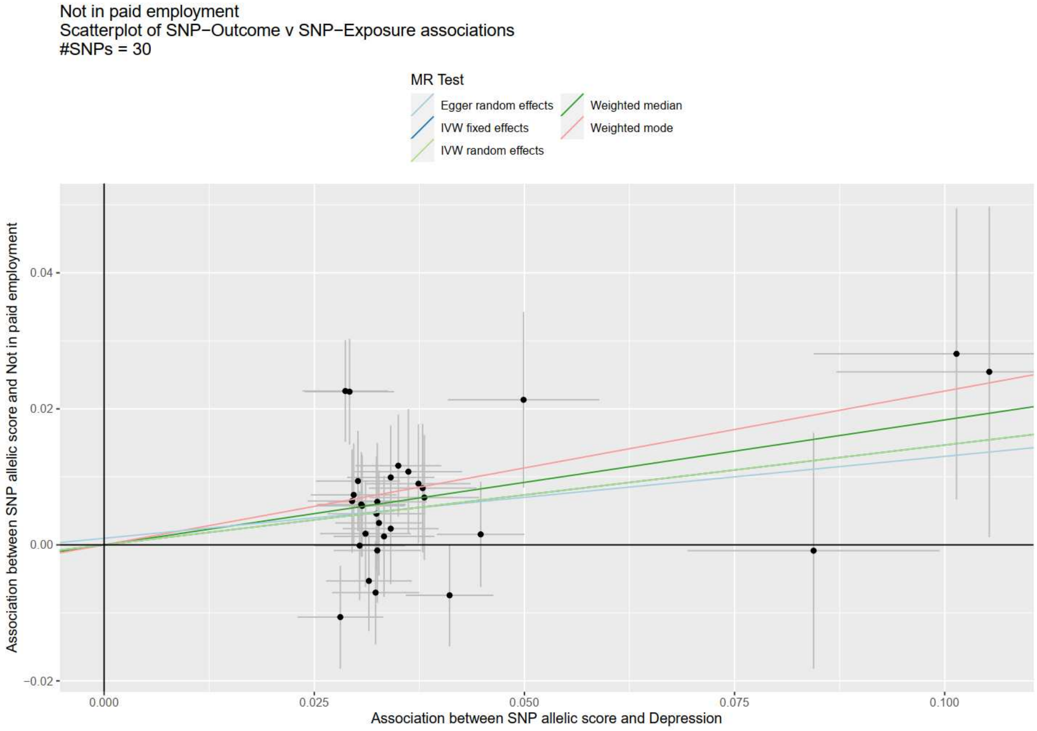

Footnote: SNP=single nucleotide polymorphism. X axis – Depression-SNP regression coefficient estimates from Howard and colleagues, Y axis – Sick/Disabled-SNP log odds from UK Biobank regressions. Also plotted are the fits for several causal effect estimation methods.

Figure S10 Quantile-Quantile plots comparing Single SNP and Leave One SNP Out causal effect estimates for depression on the Not in paid employment outcome against Gaussian distributions.

Campbell D, *et al. J Epidemiol Community Health* 2022; 76:563–571. doi: 10.1136/jech-2021-218074

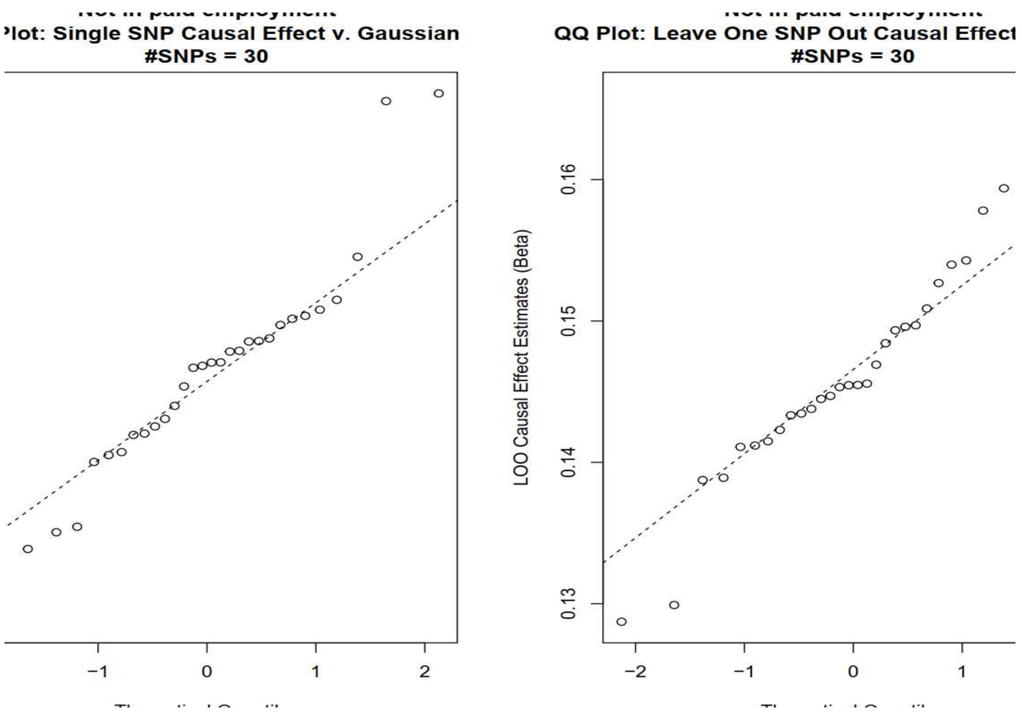

Figure S11 QQ Plots comparing SNP contribution to Cochran's Q to a Chi² df=1 distribution for Not in paid employment outcome.

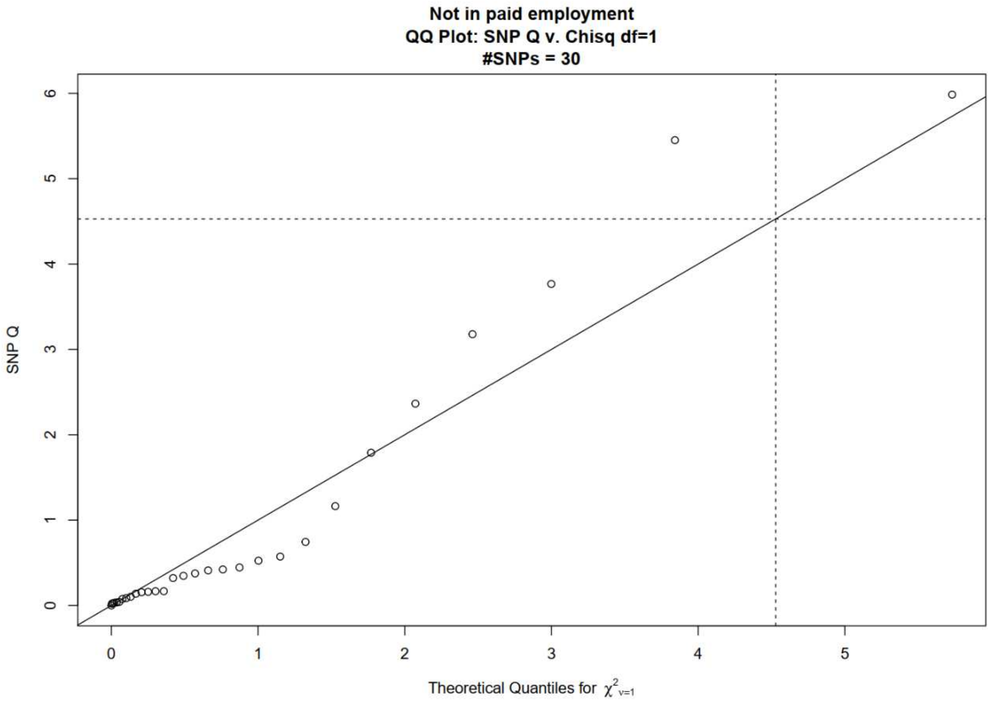

Campbell D, *et al. J Epidemiol Community Health* 2022; 76:563–571. doi: 10.1136/jech-2021-218074

Figure S12 Rücker model Selection Framework Plots for Not in paid employment outcome.

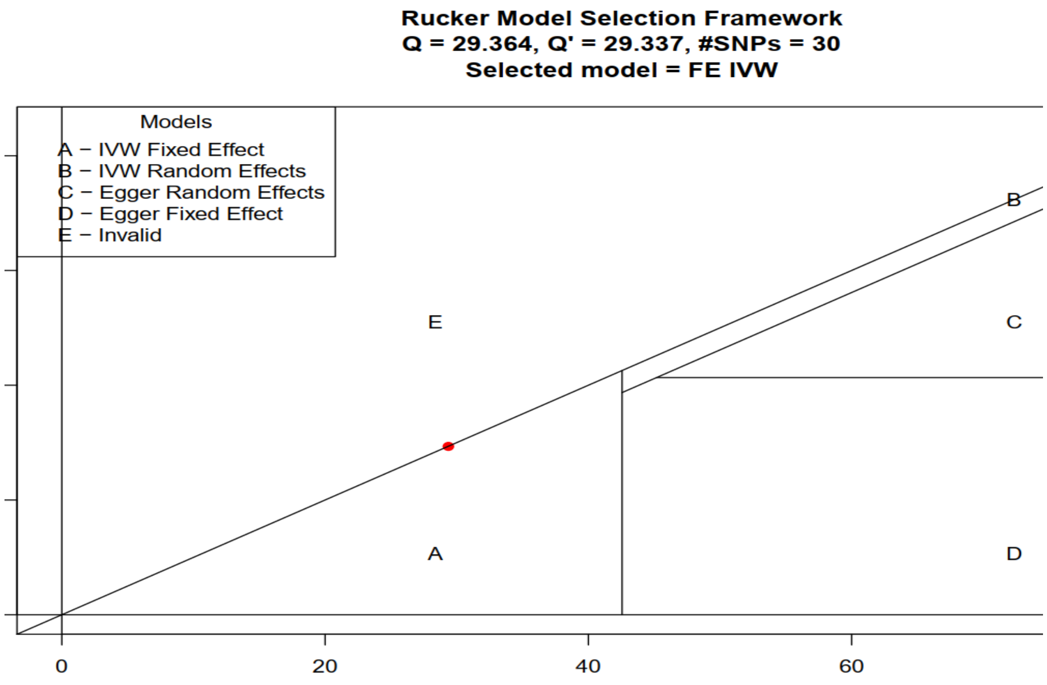

Footnote: The region in which the red dot lies indicates the model selected.

6 Tables

Table S1 Observational analyses for the association between depression and employment related outcomes

Employment Category	Regressor	Odds Ratio	Odds Ratio 95% CI	LRT P Value
Not in paid employment	Depression	2.27	(2.21, 2.33)	0.0E+00
Not in paid employment	Age	1.15	(1.15, 1.15)	0.0E+00
Not in paid employment	Assessment Centre	NA	NA	3.3E-125
Not in paid employment	Sex (=Female)	1.07	(1.04, 1.09)	6.9E-09
Not in paid employment	PGC23	0.99	(0.99, 0.998)	1.3E-03
Sick/Disabled	Depression	6.27	(6.02, 6.53)	0.0E+00
Sick/Disabled	Age	1.09	(1.09, 1.09)	0.0E+00
Sick/Disabled	Assessment Centre	NA	NA	2.2E-247
Sick/Disabled	PGC5	1.01	(1.01, 1.02)	2.6E-11
Sick/Disabled	Sex (=Female)	0.88	(0.841, 0.913)	3.3E-10
Caring for Home/Family	Sex (=Female)	7.80	(7.31, 8.33)	0.0E+00

Campbell D, *et al. J Epidemiol Community Health* 2022; 76:563–571. doi: 10.1136/jech-2021-218074

Caring for Home/Family	Depression	1.43	(1.35, 1.51)	2.0E-32
Caring for Home/Family	Assessment Centre	NA	NA	1.4E-21
Caring for Home/Family	Age	1.019	(1.02, 1.02)	1.8E-21
Caring for Home/Family	PGC5	0.99	(0.989, 0.999)	1.8E-02
Retired	Age	1.38	(1.37, 1.38)	0.0E+00
Retired	Assessment Centre	NA	NA	8.2E-62
Retired	Depression	1.38	(1.33, 1.44)	3.8E-50
Retired	PGC14	1.012	(1.01, 1.02)	3.6E-06
Retired	PGC34	1.007	(1, 1.01)	8.3E-03
Unemployed	Assessment Centre	NA	NA	5.6E-134
Unemployed	Sex (=Female)	0.51	(0.477, 0.538)	4.4E-112
Unemployed	Depression	2.13	(1.99, 2.28)	1.7E-91
Unemployed	Age	1.02	(1.02, 1.03)	6.7E-27
Unemployed	PGC14	0.98	(0.974, 0.993)	8.7E-04

Outcome	Regressor	Beta	Beta 95% CI	LRT P Value
Townsend Deprivation Index	Depression	0.80	(0.765, 0.83)	0.0E+00
Townsend Deprivation Index	Assessment Centre	NA	NA	0.0E+00
Townsend Deprivation Index	Age	-0.03	(-0.0299, -0.0264)	1.1E-212
Townsend Deprivation Index	PGC5	0.03	(0.0227, 0.0279)	3.5E-82
Townsend Deprivation Index	Sex (=Female)	-0.15	(-0.171, -0.124)	2.2E-34
Hours Worked	Age	-0.24	(-0.244, -0.227)	0.0E+00
Hours Worked	Sex (=Female)	-8.54	(-8.65, -8.43)	0.0E+00
Hours Worked	Assessment Centre	NA	NA	2.0E-55
Hours Worked	Depression	-1.09	(-1.25, -0.927)	2.7E-39
Hours Worked	PGC5	0.040	(0.0282, 0.0524)	6.3E-11
Number in Household	Age	-0.058	(-0.0587, -0.0571)	0.0E+00
Number in Household	Depression	-0.23	(-0.245, -0.216)	2.0E-208
Number in Household	Assessment Centre	NA	NA	3.6E-172
Number in Household	Sex (=Female)	-0.048	(-0.0588, -0.0374)	1.2E-18
Number in Household	PGC25	-0.003	(-0.00493, -0.00113)	1.8E-03

Outcome	Regressor	Odds Ratio	Odds Ratio 95% CI	LRT P Value
Highest Educational Attainment	Age	0.98	(0.975, 0.977)	0.0E+00
Highest Educational Attainment	Assessment Centre	NA	NA	0.0E+00
Highest Educational Attainment	Depression	0.83	(0.815, 0.85)	3.2E-65
Highest Educational Attainment	PGC4	0.98	(0.978, 0.985)	1.2E-23
Highest Educational Attainment	PGC9	0.99	(0.99, 0.993)	8.0E-18

Outcome	Regressor	Odds Ratio	Odds Ratio 95% CI	LRT P Value
Household Income	Depression	0.53	(0.515, 0.539)	0.0E+00
Household Income	Age	0.97	(0.97, 0.973)	0.0E+00
Household Income	Assessment Centre	NA	NA	0.0E+00
Household Income	Number in Household (winsorised)	1.68	(1.67, 1.69)	0.0E+00
Household Income	Sex (=Female)	0.91	(0.891, 0.921)	1.2E-32

Campbell D, et al. J Epidemiol Community Health 2022; 76:563–571. doi: 10.1136/jech-2021-218074

Footnote: 'LRT P Value' gives the p-value for a likelihood ratio test of whether dropping the regressor degrades fit.

Table S2 Regression of exposure on polygenic score

Regressor	Estimate	95% CI	Pr(>|z|)	Df	AIC	Delta AIC	Relative Likelihood
(Intercept)	-2.91	(-3.37,-2.45)	3.2E-35	NA	NA	NA	NA
sex (=female)	0.36	(0.33,0.38)	3.6E-184	1	189858	839.5	5.0E-183
Assessment Centre	NA	NA	NA	21	189299	280.5	1.2E-61
polygenic risk score	0.0203	(0.017,0.024)	2.5E-31	1	189152	133.6	9.7E-30
PGC14	-0.0114	(-0.015,-0.007)	4.1E-08	1	189047	28.2	7.7E-07

Footnote: Regression of exposure on polygenic score plus covariates. The left half of the table gives log odds ratio regression coefficient estimates. The right half gives statistics regarding how dropping the regressor worsened fit. The table is ordered by relative likelihood. The table is truncated to the set of regressors with relative likelihood < 0.05. The sex regression coefficient estimate uses males as the reference group. Assessment Centre was coded as a set of dummy variables and therefore the regression results are displayed as NA. PGC = Principal Genetic Component.

Table S3 Causal effect estimates (2sample MR) for depression on employment status

Outcome	method	Odds Ratio	Odds Ratio 95% CI	Odds Ratio P Value	P Value < 0.05
Not in paid employment	Weighted median	1.20	(1.066, 1.354)	2.6E-03	*
Not in paid employment	Penalised weighted median	1.20	(1.065, 1.356)	2.8E-03	*
Not in paid employment	Simple mode	1.25	(0.9882, 1.58)	7.3E-02	
Not in paid employment	Weighted mode (NOME)	1.26	(0.9837, 1.608)	7.8E-02	
Not in paid employment	Simple mode (NOME)	1.25	(0.9919, 1.575)	6.9E-02	
Not in paid employment	RAPS simple robust	1.16	(1.056, 1.264)	1.6E-03	*
Not in paid employment	Weighted mode	1.25	(0.9939, 1.582)	6.6E-02	
Not in paid employment	Egger fixed effects	1.13	(0.8112, 1.569)	4.8E-01	
Not in paid employment	Egger random effects	1.13	(0.8049, 1.581)	4.9E-01	
Not in paid employment	MR Egger (bootstrap 50k)	0.95	(0.6888, 1.301)	3.7E-01	
Not in paid employment	IVW fixed effects	1.16	(1.064, 1.26)	6.7E-04	*
Not in paid employment	Simple median	1.21	(1.075, 1.362)	1.6E-03	*
Not in paid employment	Maximum likelihood	1.16	(1.066, 1.266)	6.2E-04	*
Not in paid employment	IVW random effects	1.16	(1.064, 1.261)	7.2E-04	*
Sick/Disabled	Simple median	1.61	(1.284, 2.013)	3.4E-05	*
Sick/Disabled	Weighted median	1.57	(1.262, 1.964)	5.8E-05	*
Sick/Disabled	Weighted mode	1.81	(1.136, 2.895)	1.9E-02	*

Campbell D, *et al. J Epidemiol Community Health* 2022; 76:563–571. doi: 10.1136/jech-2021-218074

Sick/Disabled	Egger fixed effects	1.33	(0.726, 2.421)	3.7E-01	
Sick/Disabled	Penalised weighted median	1.60	(1.291, 1.995)	2.1E-05	*
Sick/Disabled	RAPS simple robust	1.61	(1.363, 1.909)	2.7E-08	*
Sick/Disabled	IVW fixed effects	1.56	(1.344, 1.819)	7.4E-09	*
Sick/Disabled	IVW random effects	1.56	(1.344, 1.819)	7.4E-09	*
Sick/Disabled	Simple mode	1.58	(1.003, 2.494)	5.8E-02	
Sick/Disabled	Weighted mode (NOME)	1.87	(1.132, 3.085)	2.1E-02	*
Sick/Disabled	Simple mode (NOME)	1.58	(1.004, 2.49)	5.7E-02	
Sick/Disabled	Maximum likelihood	1.58	(1.348, 1.86)	2.1E-08	*
Sick/Disabled	Egger random effects	1.33	(0.726, 2.421)	3.7E-01	
Sick/Disabled	MR Egger (bootstrap 50k)	1.43	(0.818, 2.516)	1.0E-01	
Caring for Home/Family	Simple median	1.24	(0.9686, 1.592)	8.8E-02	
Caring for Home/Family	Weighted median	1.23	(0.9492, 1.586)	1.2E-01	
Caring for Home/Family	Penalised weighted median	1.23	(0.9466, 1.594)	1.2E-01	
Caring for Home/Family	Simple mode	1.32	(0.827, 2.117)	2.5E-01	
Caring for Home/Family	Weighted mode	1.28	(0.79, 2.087)	3.2E-01	
Caring for Home/Family	Weighted mode (NOME)	1.29	(0.797, 2.094)	3.1E-01	
Caring for Home/Family	Simple mode (NOME)	1.32	(0.7849, 2.231)	3.0E-01	
Caring for Home/Family	RAPS simple robust	1.22	(1.002, 1.475)	4.8E-02	*
Caring for Home/Family	Maximum likelihood	1.17	(0.9715, 1.406)	9.8E-02	
Caring for Home/Family	Egger fixed effects	0.83	(0.4172, 1.644)	5.9E-01	
Caring for Home/Family	Egger random effects	0.83	(0.4172, 1.644)	5.9E-01	
Caring for Home/Family	MR Egger (bootstrap 50k)	0.80	(0.4124, 1.54)	2.5E-01	
Caring for Home/Family	IVW fixed effects	1.16	(0.9769, 1.386)	9.0E-02	
Caring for Home/Family	IVW random effects	1.16	(0.9769, 1.386)	9.0E-02	
Retired	Simple median	1.02	(0.8698, 1.205)	7.8E-01	
Retired	Weighted median	1.03	(0.8695, 1.211)	7.6E-01	
Retired	Penalised weighted median	1.03	(0.8693, 1.21)	7.6E-01	
Retired	Simple mode	1.04	(0.7635, 1.405)	8.2E-01	
Retired	Maximum likelihood	1.08	(0.9586, 1.218)	2.0E-01	
Retired	Weighted mode (NOME)	1.05	(0.7905, 1.4)	7.3E-01	
Retired	Egger random effects	1.04	(0.6118, 1.764)	8.9E-01	
Retired	RAPS simple robust	1.04	(0.9169, 1.175)	5.6E-01	
Retired	IVW fixed effects	1.08	(0.9578, 1.212)	2.1E-01	
Retired	IVW random effects	1.08	(0.9429, 1.232)	2.7E-01	
Retired	Egger fixed effects	1.04	(0.6564, 1.644)	8.7E-01	
Retired	Simple mode (NOME)	1.04	(0.7512, 1.428)	8.3E-01	
Retired	Weighted mode	1.05	(0.8002, 1.383)	7.2E-01	
Retired	MR Egger (bootstrap 50k)	1.00	(0.6274, 1.596)	5.0E-01	
Unemployed	Simple median	1.14	(0.8258, 1.569)	4.3E-01	
Unemployed	Weighted median	1.07	(0.7732, 1.475)	6.9E-01	
Unemployed	Simple mode (NOME)	1.57	(0.7705, 3.207)	2.2E-01	
Unemployed	MR Egger (bootstrap 50k)	0.75	(0.324, 1.721)	2.5E-01	
Unemployed	IVW fixed effects	1.03	(0.8403, 1.266)	7.7E-01	
Unemployed	IVW random effects	1.03	(0.8403, 1.266)	7.7E-01	
Unemployed	Penalised weighted median	1.07	(0.766, 1.489)	7.0E-01	
Unemployed	Simple mode	1.57	(0.7906, 3.125)	2.1E-01	
Unemployed	Weighted mode	1.53	(0.7734, 3.026)	2.3E-01	

Unemployed	Weighted mode (NOME)	1.55	(0.7474, 3.218)	2.5E-01
Unemployed	RAPS simple robust	1.04	(0.8172, 1.328)	7.4E-01
Unemployed	Maximum likelihood	1.03	(0.8184, 1.301)	7.9E-01
Unemployed	Egger fixed effects	0.75	(0.3393, 1.653)	4.8E-01
Unemployed	Egger random effects	0.75	(0.3393, 1.653)	4.8E-01

Table S4 Causal effect estimates (2sample MR) for depression on TDI and Weekly Hours Worked

Outcome	method	Beta	Beta 95% CI	Beta P Value	P Value < 0.05
Townsend Deprivation Index	Simple median	0.158	(-0.010, 0.326)	6.6E-02	
Townsend Deprivation Index	Weighted median	0.176	(0.017, 0.334)	3.0E-02	*
Townsend Deprivation Index	Penalised weighted median	0.256	(0.097, 0.414)	1.6E-03	*
Townsend Deprivation Index	Simple mode	0.324	(-0.055, 0.703)	1.0E-01	
Townsend Deprivation Index	Weighted mode	0.301	(-0.0017, 0.61)	6.1E-02	
Townsend Deprivation Index	Weighted mode (NOME)	0.324	(-0.0317, 0.68)	8.5E-02	
Townsend Deprivation Index	Simple mode (NOME)	0.324	(-0.097, 0.75)	1.4E-01	
Townsend Deprivation Index	RAPS simple robust	0.163	(0.065, 0.26)	1.1E-03	*
Townsend Deprivation Index	RAPS overdispersed robust	0.058	(-0.126, 0.242)	5.4E-01	
Townsend Deprivation Index	Maximum likelihood	0.097	(-0.0012, 0.20)	5.3E-02	
Townsend Deprivation Index	Egger fixed effects	-0.203	(-0.571, 0.165)	2.9E-01	
Townsend Deprivation Index	Egger random effects	-0.203	(-0.866, 0.459)	5.5E-01	
Townsend Deprivation Index	MR Egger (bootstrap 50k)	0.214	(-0.24, 0.667)	1.8E-01	
Townsend Deprivation Index	IVW fixed effects	0.095	(0.00048, 0.19)	4.9E-02	*
Townsend Deprivation Index	IVW random effects	0.095	(-0.074, 0.26)	2.7E-01	
Hours Worked	Weighted median	-0.264	(-0.93, 0.401)	4.4E-01	
Hours Worked	Simple mode	-0.284	(-1.37, 0.797)	6.1E-01	
Hours Worked	Simple median	-0.235	(-0.87, 0.40)	4.7E-01	
Hours Worked	Weighted mode (NOME)	-0.284	(-1.36, 0.7892)	6.1E-01	
Hours Worked	Penalised weighted median	-0.245	(-0.89, 0.396)	4.5E-01	
Hours Worked	RAPS simple robust	-0.082	(-0.54, 0.3788)	7.3E-01	
Hours Worked	RAPS overdispersed robust	-0.072	(-0.627, 0.484)	8.0E-01	
Hours Worked	Maximum likelihood	-0.127	(-0.577, 0.323)	5.8E-01	
Hours Worked	Egger fixed effects	0.310	(-1.41, 2.03)	7.3E-01	
Hours Worked	Egger random effects	0.310	(-1.886, 2.51)	7.8E-01	
Hours Worked	MR Egger (bootstrap 50k)	-0.518	(-2.3, 1.27)	2.8E-01	
Hours Worked	IVW fixed effects	-0.130	(-0.57, 0.31)	5.6E-01	
Hours Worked	IVW random effects	-0.130	(-0.685, 0.424)	6.5E-01	
Hours Worked	Weighted mode	-0.284	(-1.294, 0.727)	5.9E-01	
Hours Worked	Simple mode (NOME)	-0.284	(-1.50, 0.934)	6.5E-01	

Table S5 Causal effect estimates (2sample MR) for depression on Household Income Level and Highest Educational Attainment

Outcome	method	Odds	Odds	Odds	P Value

Campbell D, et al. J Epidemiol Community Health 2022; 76:563–571. doi: 10.1136/jech-2021-218074

		Ratio	Ratio 95% CI	Ratio P Value	< 0.05
Highest Educational Attainment	Simple median	0.99	(0.8908, 1.096)	8.2E-01	
Highest Educational Attainment	Weighted median	0.99	(0.8863, 1.098)	8.1E-01	
Highest Educational Attainment	Penalised weighted median	0.96	(0.8649, 1.068)	4.6E-01	
Highest Educational Attainment	Simple mode	0.95	(0.7992, 1.14)	6.1E-01	
Highest Educational Attainment	Weighted mode	0.97	(0.8238, 1.136)	6.9E-01	
Highest Educational Attainment	Weighted mode (NOME)	0.97	(0.8205, 1.14)	7.0E-01	
Highest Educational Attainment	Simple mode (NOME)	0.95	(0.8057, 1.131)	5.9E-01	
Highest Educational Attainment	RAPS simple robust	1.00	(0.9439, 1.064)	9.4E-01	
Highest Educational Attainment	RAPS overdispersed robust	0.98	(0.8445, 1.127)	7.4E-01	
Highest Educational Attainment	Maximum likelihood	1.11	(1.04, 1.189)	2.0E-03	*
Highest Educational Attainment	Egger fixed effects	0.89	(0.6982, 1.123)	3.2E-01	
Highest Educational Attainment	Egger random effects	0.89	(0.4859, 1.613)	6.9E-01	
Highest Educational Attainment	MR Egger (bootstrap 50k)	1.72	(1.196, 2.48)	2.5E-03	*
Highest Educational Attainment	IVW fixed effects	1.09	(1.028, 1.161)	4.4E-03	*
Highest Educational Attainment	IVW random effects	1.09	(0.9378, 1.273)	2.6E-01	
Household Income	Simple median	0.88	(0.7858, 0.976)	1.6E-02	*
Household Income	Weighted median	0.96	(0.8548, 1.075)	4.7E-01	
Household Income	Penalised weighted median	0.88	(0.7843, 0.9807)	2.1E-02	*
Household Income	Simple mode	0.81	(0.6071, 1.074)	1.5E-01	
Household Income	Weighted mode	0.82	(0.5836, 1.161)	2.8E-01	
Household Income	Weighted mode (NOME)	0.81	(0.5683, 1.165)	2.7E-01	
Household Income	Simple mode (NOME)	0.81	(0.6122, 1.065)	1.4E-01	
Household Income	RAPS simple robust	0.93	(0.8696, 0.9941)	3.3E-02	*
Household Income	RAPS overdispersed robust	0.96	(0.8675, 1.066)	4.6E-01	
Household Income	Maximum likelihood	0.97	(0.9053, 1.035)	3.4E-01	
Household Income	Egger fixed effects	1.24	(0.9633, 1.595)	1.1E-01	
Household Income	Egger random effects	1.24	(0.8168, 1.881)	3.2E-01	
Household Income	MR Egger (bootstrap 50k)	1.33	(0.9989, 1.783)	2.8E-02	*
Household Income	IVW fixed effects	0.97	(0.9087, 1.034)	3.5E-01	
Household Income	IVW random effects	0.97	(0.8704, 1.08)	5.7E-01	

Table S6 Heterogeneity tests

Outcome	Method	Q	Q df	Q P Value	P Value < 0.05
Not in paid employment	Inverse variance weighted	29.36	29	4.5E-01	
Not in paid employment	IVW radial	29.07	29	4.6E-01	
Not in paid employment	Maximum likelihood	29.18	29	4.6E-01	
Not in paid employment	MR Egger	29.34	28	4.0E-01	
Not in paid employment	Unweighted regression	0.00	29	1.0E+00	
Sick/Disabled	Inverse variance weighted	27.04	29	5.7E-01	
Sick/Disabled	IVW radial	26.28	29	6.1E-01	
Sick/Disabled	Maximum likelihood	26.63	29	5.9E-01	
Sick/Disabled	MR Egger	26.75	28	5.3E-01	
Sick/Disabled	Unweighted regression	0.01	29	1.0E+00	

Campbell D, *et al. J Epidemiol Community Health* 2022; 76:563–571. doi: 10.1136/jech-2021-218074

Caring for Home/Family	Inverse variance weighted	26.61	29	5.9E-01	
Caring for Home/Family	IVW radial	26.55	29	6.0E-01	
Caring for Home/Family	Maximum likelihood	26.57	29	6.0E-01	
Caring for Home/Family	MR Egger	25.69	28	5.9E-01	
Caring for Home/Family	Unweighted regression	0.01	29	1.0E+00	
Retired	Inverse variance weighted	37.27	29	1.4E-01	
Retired	IVW radial	37.22	29	1.4E-01	
Retired	Maximum likelihood	37.25	29	1.4E-01	
Retired	MR Egger	37.25	28	1.1E-01	
Retired	Unweighted regression	0.01	29	1.0E+00	
Unemployed	Inverse variance weighted	23.12	29	7.7E-01	
Unemployed	IVW radial	23.12	29	7.7E-01	
Unemployed	Maximum likelihood	23.12	29	7.7E-01	
Unemployed	MR Egger	22.58	28	7.5E-01	
Unemployed	Unweighted regression	0.02	29	1.0E+00	
Hours Worked	Inverse variance weighted	46.00	29	2.3E-02	*
Hours Worked	IVW radial	45.99	29	2.4E-02	*
Hours Worked	Maximum likelihood	45.99	29	2.4E-02	*
Hours Worked	MR Egger	45.73	28	1.9E-02	*
Hours Worked	Unweighted regression	0.11	29	1.0E+00	
Townsend Deprivation Index	Inverse variance weighted	93.35	29	1.1E-08	*
Townsend Deprivation Index	IVW radial	93.03	29	1.2E-08	*
Townsend Deprivation Index	Maximum likelihood	93.07	29	1.2E-08	*
Townsend Deprivation Index	MR Egger	90.65	28	1.6E-08	*
Townsend Deprivation Index	Unweighted regression	0.01	29	1.0E+00	
Highest Educational Attainment	Inverse variance weighted	181.99	29	4.3E-24	*
Highest Educational Attainment	IVW radial	180.68	29	7.5E-24	*
Highest Educational Attainment	Maximum likelihood	180.66	29	7.6E-24	*
Highest Educational Attainment	MR Egger	178.76	28	6.6E-24	*
Highest Educational Attainment	Unweighted regression	0.01	29	1.0E+00	
Household Income	Inverse variance weighted	80.53	29	9.7E-07	*
Household Income	IVW radial	80.47	29	9.9E-07	*
Household Income	Maximum likelihood	80.49	29	9.8E-07	*
Household Income	MR Egger	76.62	28	2.1E-06	*
Household Income	Unweighted regression	0.00	29	1.0E+00	

Footnote: Heterogeneity test results. Q – test statistic, Q df – degrees of freedom, P Value – p-value for null hypothesis of no heterogeneity across SNPs in causal effect size estimate.

Table S7 Unbalanced pleiotropy tests

Outcome	Method	Egger intercept	Std Err	P Value	P Value < 0.05
Not in paid employment	MR Egger	0.00097	0.0061	0.88	
Sick/Disabled	MR Egger	0.00604	0.0111	0.59	

Caring for Home/Family	MR Egger	0.01247	0.0129	0.34
Retired	MR Egger	0.00135	0.0096	0.89
Unemployed	MR Egger	0.01178	0.0160	0.47
Hours Worked	MR Egger	-0.01618	0.0398	0.69
Townsend Deprivation Index	MR Egger	0.01095	0.0120	0.37
Highest Educational Attainment	MR Egger	0.00772	0.0109	0.48
Household Income	MR Egger	-0.00902	0.0075	0.24

Table S8 Rücker Model Selection Framework

Outcome	Cochrans Q	Rückers Q	Selected Model
Not in paid employment	29.36	29.34	IVW fixed effects
Sick/Disabled	27.04	26.75	IVW fixed effects
Caring for Home/Family	26.61	25.69	IVW fixed effects
Retired	37.27	37.25	IVW fixed effects
Unemployed	23.12	22.58	IVW fixed effects
Townsend Deprivation Index	93.35	90.65	IVW random effects
Hours Worked	46.00	45.73	IVW random effects
Highest Educational Attainment	181.99	178.76	IVW random effects
Household Income	80.53	76.62	Egger random effects

Footnote: The less parsimonious model was rejected at a p-value of > 0.05.

Table S9 Tests for sex difference in causal effect estimates

Outcome	MR Method	Rücker Model Male	Rücker Model Female	OR Male	OR 95% CI Male	OR Female	OR 95% CI Female	Sex Difference P Value
Not in paid employment	IVW fixed effects	Y	Y	1.16	(1.04, 1.3)	1.17	(1.04, 1.31)	0.98
Sick/Disabled	IVW fixed effects	Y	Y	1.50	(1.25, 1.79)	1.67	(1.31, 2.12)	0.47
Caring for Home/Family	IVW fixed effects	Y	Y	1.28	(0.831, 1.96)	1.15	(0.951, 1.39)	0.66
Retired	IVW fixed effects	Y	Y	1.13	(0.981, 1.3)	0.96	(0.785, 1.19)	0.22
Unemployed	IVW fixed effects	Y	Y	1.07	(0.829, 1.39)	0.93	(0.641, 1.36)	0.55

Outcome	MR Method	Rücker Model Male	Rücker Model Female	Beta Male	Beta 95% CI Male	Beta Female	Beta 95% CI Female	Sex Difference P Value
Townsend Deprivation Index	IVW random effects	Y	Y	0.121	(-0.0845, 0.327)	0.060	(-0.163, 0.284)	0.70
Hours Worked	IVW fixed effects	Y		0.045	(-0.545, 0.635)	-0.379	(-1.03, 0.276)	0.35
Hours Worked	IVW random effects		Y	0.045	(-0.609, 0.699)	-0.379	(-1.17, 0.417)	0.42

Outcome	MR	Rücker	Rücker	OR	OR	OR	OR	Sex

Campbell D, *et al. J Epidemiol Community Health* 2022; 76:563–571. doi: 10.1136/jech-2021-218074

	Method	Model Male	Model Female	Male	95% CI Male	Female	95% CI Female	Difference P Value
Highest Educational Attainment	IVW random effects	Y	Y	1.06	(0.913, 1.22)	1.14	(0.945, 1.37)	0.53
Household Income	IVW random effects	Y	Y	0.95	(0.85, 1.06)	0.99	(0.868, 1.14)	0.60

Table S10 UK Biobank Fields used in the study

UK Biobank field	Type	Description	Comment
**QC Related**			
21000	categorical	Ethnic background	An amalgam of sequential branching questions asked during the initial Assessment Centre visit
22006	dichotomous	Genetic ethnic grouping	Indicates samples who self-identified as 'White British' according to Field 21000 and have very similar genetic ancestry based on a principal components analysis of the genotypes.
22019	dichotomous	Sex chromosome aneuploidy	Sex chromosome aneuploidy marker. This indicates samples which were identified as putatively carrying sex chromosome configurations that are not either XX or XY.
22027	dichotomous	Outliers for heterozygosity or missing rate	Indicates samples identified as outliers in heterozygosity and missing rates, which implies that the genotypes for these samples are of poor quality.
22005	continuous	Missingness	Missing rate of each sample based on a set of high-quality markers.
22001	dichotomous	Genetic sex	Sex as determined from genotyping analysis.
31	dichotomous	Sex	Sex of participant. Acquired from central registry at recruitment, but in some cases updated by the participant.
22009	continuous	Genetic principal components	Score for each principal component 1-40
54	categorical	UK Biobank Assessment Centre	UK Biobank assessment centre at which participant consented, 1 of 22 centres
22021	continuous	Genetic kinship to other participants	A threshold lowest kinship coefficient of 0.042 (equivalent to second cousins) was used to identify overly related pairs of participants
**Outcome Related**			
6142	categorical	Current employment status was self-reported	
767	continuous	Length of working week for main job	Collected from all the participants who indicated they were in paid employment or self-employed (see Field 6142)

Campbell D, *et al. J Epidemiol Community Health* 2022; 76:563–571. doi: 10.1136/jech-2021-218074

189	continuous	Townsend deprivation index at recruitment	Townsend deprivation index calculated immediately prior to participant joining UK Biobank.
738	ordinal	Average total household income before tax	Collected from participants except those who indicated they were living in a sheltered accommodation or in a care home
6138	ordinal	Education Qualifications	
**Exposure Related**			
20002	categorical	Non-cancer illness code, self-reported	Depression indicated by code = 1286
2100	dichotomous	Seen a psychiatrist for nerves, anxiety, tension or depression	
2090	dichotomous	Seen doctor (GP) for nerves, anxiety, tension or depression	not used
41202	categorical	Diagnoses - main ICD10	Depression indicated by codes matching 'F32*' and 'F33*'
41204	categorical	Diagnoses - secondary ICD10	not used
41203	categorical	Diagnoses - main ICD9	Depression indicated by codes matching '296*' or '311*'
41205	categorical	Diagnoses - secondary ICD9	not used

Table S11 Instrument SNP set used in the study

SNP	Chr	Effect allele	Other allele	Beta	Std Err	P Value	Effect allele freq
rs1432639	1	A	C	0.041	0.0052	4.6E-15	0.63
rs2332571	1	T	C	0.033	0.006	3.2E-08	0.23
rs10913112	1	T	C	-0.030	0.0053	2.2E-08	0.38
rs4660091	1	T	C	-0.029	0.0053	3.7E-08	0.66
rs159963	1	A	C	-0.030	0.0051	6.9E-09	0.55
rs12129573	1	A	C	0.033	0.0052	3.7E-10	0.37
rs11682175	2	T	C	-0.029	0.0051	1.4E-08	0.52
rs76485002	2	A	G	0.105	0.0182	7.2E-09	0.97
rs7430565	3	A	G	-0.031	0.0051	1.7E-09	0.58
rs34215985	4	C	G	-0.036	0.0064	1.3E-08	0.24
rs75990749	4	C	G	0.084	0.015	1.9E-08	0.04
rs12658032	5	A	G	0.031	0.0053	8.6E-09	0.36
rs11135349	5	A	C	-0.032	0.0051	5.0E-10	0.48
rs10044618	5	T	C	0.035	0.0051	7.3E-12	0.45
rs12202410	6	A	T	-0.033	0.0059	3.3E-08	0.76

Campbell D, *et al. J Epidemiol Community Health* 2022; 76:563–571. doi: 10.1136/jech-2021-218074

rs6456727	6	T	C	0.038	0.0065	5.1E-09	0.81
rs77457816	9	A	G	0.101	0.017	2.6E-09	0.03
rs7036618	9	A	C	0.028	0.0051	4.5E-08	0.59
rs61867293	10	T	C	-0.038	0.0064	2.8E-09	0.20
rs1806153	11	T	G	0.037	0.0062	2.2E-09	0.22
rs1343605	13	A	C	0.045	0.0052	6.3E-18	0.39
rs1950829	14	A	G	0.030	0.005	1.8E-09	0.49
rs12886138	14	T	C	-0.032	0.0052	6.7E-10	0.39
rs632123	15	T	C	-0.034	0.0052	6.0E-11	0.63
rs7200826	16	T	C	0.032	0.0058	2.5E-08	0.25
rs7198928	16	T	C	0.033	0.0052	4.4E-10	0.62
rs8063603	16	A	G	-0.030	0.0054	1.6E-08	0.65
rs77135925	17	T	C	-0.050	0.009	3.4E-08	0.92
rs12958048	18	A	G	0.031	0.0054	7.1E-09	0.33
rs5758265	22	A	G	0.034	0.0057	1.8E-09	0.28

7 References

1. Howard DM, Adams MJ, Clarke TK, et al. Genome-wide meta-analysis of depression identifies 102 independent variants and highlights the importance of the prefrontal brain regions. *Nat Neurosci* 2019;22(3):343-52. doi: 10.1038/s41593-018-0326-7 [published Online First: 2019/02/06]
2. Chang CC, Chow CC, Tellier LC, et al. Second-generation PLINK: rising to the challenge of larger and richer datasets. *Gigascience* 2015;4:7. doi: 10.1186/s13742-015-0047-8 [published Online First: 2015/02/28]
3. Kessler RC, Berglund P, Demler O, et al. Lifetime prevalence and age-of-onset distributions of DSM-IV disorders in the National Comorbidity Survey Replication. *Arch Gen Psychiatry* 2005;62(6):593-602. doi: 10.1001/archpsyc.62.6.593 [published Online First: 2005/06/09]
4. Hemani G, Zheng J, Elsworth B, et al. The MR-Base platform supports systematic causal inference across the human phenome. *Elife* 2018;7 doi: 10.7554/eLife.34408 [published Online First: 2018/05/31]
5. Rucker G, Schwarzer G, Carpenter JR, et al. Treatment-effect estimates adjusted for small-study effects via a limit meta-analysis. *Biostatistics* 2011;12(1):122-42. doi: 10.1093/biostatistics/kxq046 [published Online First: 2010/07/27]
6. Bowden J, Spiller W, Del Greco MF, et al. Improving the visualization, interpretation and analysis of two-sample summary data Mendelian randomization via the Radial plot and Radial regression. *Int J Epidemiol* 2018;47(4):1264-78. doi: 10.1093/ije/dyy101 [published Online First: 2018/07/03]
7. Hyde CL, Nagle MW, Tian C, et al. Identification of 15 genetic loci associated with risk of major depression in individuals of European descent. *Nat Genet* 2016;48(9):1031-6. doi: 10.1038/ng.3623 [published Online First: 2016/08/02]
8. Wray NR, Ripke S, Mattheisen M, et al. Genome-wide association analyses identify 44 risk variants and refine the genetic architecture of major depression. *Nat Genet* 2018;50(5):668-81. doi: 10.1038/s41588-018-0090-3 [published Online First: 2018/04/28]
